# Mapping Arsenic Risks in the Ayeyarwady (Irrawaddy) Delta, Myanmar: Implications for Public Health

**DOI:** 10.1029/2024GH001326

**Published:** 2025-10-29

**Authors:** M. A. Hoque, K. K. Khaing, M. Fowler, M. S. Sultana, C. C. Myint, A. Swe, P. Dennis, S. Shahid, G. R. Fones

**Affiliations:** ^1^ School of the Environment and Life Sciences (SELS) University of Portsmouth Portsmouth UK; ^2^ Institute of the Earth and Environment University of Portsmouth Portsmouth UK; ^3^ Department of Geography Hinthada University Hinthada Myanmar; ^4^ Department of Geography University of Yangon Yangon Myanmar; ^5^ School of Environmental Sciences University of East Anglia Norwich UK; ^6^ Department of Water & Environmental Engineering Faculty of Civil Engineering Universiti Teknologi Malaysia (UTM) Johor Bahru Malaysia

**Keywords:** arsenic contamination, hydrological influences, drainage density, Ayeyarwady delta, groundwater quality, Myanmar

## Abstract

The Ayeyarwady Delta in Myanmar, home to an estimated 12 million people, faces widespread arsenic contamination similar to other Asian deltas namely Bengal, Red River, and Mekong. Arsenic here primarily results from reductive dissolution of iron minerals in anoxic conditions driven by organic carbon. Here, we used digital elevation model (DEM) data to investigate how drainage density and hierarchical recharge pathways influence arsenic distribution, supported by combined data set of 136 wells (81 new, 55 from a prior study)—up to 215 m deep—along a 170 km west‐to‐east transect across the delta. Findings indicate arsenic hotspots in the mid‐central region of the delta, where high drainage density appears to facilitate focused recharge, delivering organic carbon to underlying aquifers. Compared with other deltaic regions across Asia, the Ayeyarwady has fewer high‐arsenic wells, with only 21% of our data set exceeding the local 50 μg/l limit. National screening data from 123,962 wells indicate that while only 8% exceed the regulatory limit of 50 μg/l set by Myanmar, 71% exceed the 10 μg/l guideline recommended by the World Health Organization (WHO). This highlights widespread exposure risk not addressed under the current national standard, particularly for rural communities. The observed variability in arsenic concentrations, driven by complex redox dynamics and groundwater flow patterns, indicates that contamination can occur even within short spatial intervals. A blanket‐screening program focused on hotspot regions is essential to ensure that at‐risk populations are not unknowingly exposed to unsafe drinking water.

## Plain Language Summary

Millions of people in the Ayeyarwady Delta, Myanmar, rely on groundwater for drinking, but much of it contains arsenic, a carcinogen that can harm health. Arsenic is released from subsurface sediments through natural processes when mineral break down in low‐oxygen conditions with organic matters. Our study examined how the landscape influences arsenic contamination underground. By testing well water across the delta and using mapping techniques, we identified areas at higher risk of arsenic contamination. These “hotspots” are often found in regions with dense networks of small streams, which transport organic matters underground and trigger arsenic release. Although fewer wells in this delta exceed local safety standards compared to other regions, many do not meet stricter global guidelines. Our findings highlight the need for targeted testing in high‐risk areas to protect public health and provide safer water sources.

## Key Points

- High drainage density supports pathways for organic carbon, driving arsenic hotspots in the Ayeyarwady Delta Myanmar, South‐East Asia
- Seventy‐one percent of wells in the study area exceed WHO arsenic guidelines, posing significant health risks to rural communities
- Comprehensive arsenic screening in identified hotspots is crucial to mitigate public health threats

## 1 Introduction

Groundwater arsenic contamination represents a significant public health concern worldwide, with Southeast Asia being a focal point due to its populous and extensive deltaic regions (Fan et al., 2024; Fendorf et al., 2010). In Myanmar, the Ayeyarwady Delta is particularly vulnerable, mirroring issues observed in other Asian deltas such as the Bengal, Mekong, and Red River deltas, where arsenic in groundwater has led to considerable morbidity and mortality (Ledford, 2008; van Geen et al., 2014; Winkel et al., 2008). Most rural people in Myanmar still rely on untreated surface and rainwater, which once was the common practice throughout Southeast Asia causing widespread water‐borne diseases (Hoque et al., 2016). The rural water supplies, used by the majority of the population (ca. 65% of 60 million), are therefore crucial both for the economic development of the country and for safeguarding health and wellbeing of the rural inhabitants (Tripartite Core Group, 2008). Myanmar has not harnessed groundwater supplies, likely because of its extensive rainfall, while other Asian counterparts achieved the Millennium Development Goals (MDGs) on safe water by relying on this pathogen‐free resource (Bacquart et al., 2015; Hutton, 2012). However, most of these countries inadvertently exposed some of their population to excessive levels of carcinogenic arsenic (Ravenscroft et al., 2009), and occasionally to high levels of sodium‐salinity in coastal areas (Islam et al., 2019; Khan et al., 2011; Scheelbeek et al., 2017; Tsai et al., 2024). This was mostly due to lack of understanding of the water quality and aquifer framework at the time of groundwater development. Currently, most of the affected countries are using improved understanding to combat the arsenic problem and provide rural water supplies from the safe‐zones of the same aquifer systems (Hoque et al., 2011, 2014; Mukherjee et al., 2023; Ravenscroft et al., 2013).

Geologically, the Ayeyarwady Delta, which spreads over three administrative regions and homes ca. 12 million people, is similar to other Asian deltas with regard to arsenic concentrations in groundwater (Kravtsova et al., 2009). The ongoing government program of “safe water for all by 2030” may be in jeopardy if the occurrences of unsafe arsenic levels in tubewells and areas that are at‐risk due to sparse data are not curtailed promptly. The water sector assessment and road map report by the Asian Development Bank (ADB, 2013) identified the lack of background information and quantitative data for Myanmar as a serious constraint for effective and efficient development in the water sector. Predictions based on global land surface models (Winkel et al., 2008) and subsequent in situ testing (van Geen et al., 2014) suggested that parts of Myanmar, including the Ayeyarwady Delta, face significant arsenic contamination in groundwater. However, these assessments lacked detailed input on the aquifer framework of the region, which remains poorly constrained. In 2005, a large Arsenic Mitigation Project was set up as a partnership between the WHO, UNICEF, and WRUD that tested nearly 125,000 water points for the delta area alone. Nearly 70% of samples tested are above the WHO (10 μg/L) limit, but less than 10% are above Myanmar's national limit (50 μg/L) (Phyu, 2019). In 2001, Save the Children conducted a survey of 1,912 tube wells across 327 villages in the Ayeyarwady Delta. The wells were relatively shallow (all <90 m) and mostly located in alluvial deposits. The survey found that 45% of samples had arsenic concentrations exceeding the WHO limit (Tun, 2003).

It is now accepted that in these arsenic‐affected deltas, arsenic is released mainly because of anoxic microbial processes where solid‐phase iron and manganese (oxy)hydroxides (Fe‐MnOOH) acts as an electron acceptor and organic matter serves as the electron donor, mobilizing the arsenic that was adsorbed onto these Fe‐MnOOH (Ghosh et al., 2021; Glodowska et al., 2020; Lovley & Anderson, 2000; Nickson et al., 1998; Weber et al., 2006). This process is mediated by heterotrophic iron‐reducing bacteria. While this biogeochemical mechanism is understood, its activation in the aquifer is fundamentally linked to the hydrogeological delivery of reactive organic matter.

However, critical uncertainties remain about two key aspects of this process: (a) the source and reactivity of the organic matter that fuels iron reduction, and (b) the precise stratigraphic or depth intervals within the aquifer system where arsenic is being released. There is a growing body of studies showing that surface‐derived recharge water can facilitate the release, which suggests surficial origin of organic matter inputs (Harvey, Swartz, et al., 2002; Hoque et al., 2017; Kumar et al., 2021; Neumann et al., 2010; Wallis et al., 2020). This implies that modern, labile carbon introduced via recharge pathways may drive redox transformations in shallow aquifer zones. The role of groundwater flow is also important and it appears that arsenic mobilization is more likely to occur within the shallower part of the system (Connolly et al., 2022; Covatti et al., 2023; Donselaar et al., 2017; Ghosh & Donselaar, 2023; Hoque et al., 2017; Wallis et al., 2020).

Given these gaps, we hypothesize that understanding the spatial variability of recharge structure—particularly where and how modern recharge introduces reactive organic matter—is key to identifying potential arsenic hotspots. This knowledge would support more targeted mitigation strategies by linking hydrogeological setting to geochemical risk.

One such consideration is that reactive organic matter coming with recharge may fuel (amorphous) iron reduction, maintain the reducing conditions, or both—and may be a critical driver. Prediction of recharge areas potentially supplying reactive organic matter could therefore allow prediction of areas at risk of high‐arsenic groundwater. Our interpretation of recharge and indirect control of the surface processes is influenced by previous studies which produced large scale prediction (Amini et al., 2008; Fan et al., 2024; Nath et al., 2022; Winkel et al., 2008). In this context, the term “surface processes” refers to the combined effects of river flow, sediment deposition, and organic matter dynamics that govern the availability of carbon and oxygen in the shallow subsurface. These processes shape the geomorphological and hydrological framework of the delta, influencing groundwater recharge and flow patterns. The inherent heterogeneity of Holocene fluvial‐deltaic deposits, combined with variable porosity and permeability, creates conditions that drive the spatial variability of arsenic. While detailed, three‐dimensional hydro‐sedimentological data are unavailable in Myanmar, our approach leverages existing data and geomorphological proxies to provide insights into arsenic risk.

The aim of this study is to enhance our understanding of the three‐dimensional spatial variabilities of arsenic within the Ayeyarwady Delta. We seek to predict the distribution of arsenic across the delta, identify potential hotspots of contamination, and explore the underlying reasons for the delta's comparatively low arsenic levels relative to other Southeast Asian deltas. This comprehensive approach is designed to inform effective prevention and mitigation strategies, leveraging insights gained from similar contexts across Asia.

## 2 Study Area Description

The Ayeyarwady River—cuts through Myanmar from north to south—before reaching the sea, splits into 11 main channels and forms a large, fertile southward‐slopping delta (Chen et al., 2020). It covers an area of ∼55,000 km^2^, flanked by the Indo‐Burman and Bago Yoma (Pegu Yoma) mountain ranges in the west and east respectively (Giosan et al., 2018). This tectonically dynamic ∼900 km long intermountain landscape is the result of a progressive southward infilling of the Ayeyawady River since the Miocene, and the current delta is the latest in a series comprising this southward moving Ayeyawady depocenter (Jonell et al., 2022). The westernmost tributary of this flat delta is the Pathein‐Bassein River, while the easternmost delta boundary is the left bank of the Yangon River. The landform near the delta apex at ∼18°N, some 300 km north of the coast, exhibits alluvial ridges above the floodplain. Active and paleo‐meander belts terminate in the mid‐delta where the discharge is split to lower‐order distributary channels. Over 90% of the discharge at the delta occurs between May and October, accompanied by ∼400 Mt/yr of sediment (Chen et al., 2020; Kravtsova et al., 2009).

The delta contains fluvio‐deltaic sediments, with fluvial components diminishing toward the south where delta‐front and estuarine deposits make bulk of the heterolithic, unconsolidated sedimentary column. The delta appears to be vertically stable and has accumulated ∼40 m sediments over the last 9k yr, when the global sea level was ca. −30 m b.s.l (Giosan et al., 2018). The lithologies comprise unconsolidated sands, gravels, and clays arranged in a locally multi‐aquifer, laterally discontinuous system. The distribution of silt‐clay layers is similar to the inter‐leaved pattern described in the Bengal delta by Hoque et al. (2017), which most likely facilitates a comparable hierarchical groundwater flow (Michael & Voss, 2009).

Around 40% of households in the delta use a low‐yielding motorized pump to extract water for drinking and other household purposes, in contrast to other Asian deltas where hand pumps are the dominant water infrastructure. However, the depth of the water well in the majority cases is ∼<100 m as for the other deltas.

## 3 Approach and Methods

### 3.1 Approach—Recharge Structure as an Indicator of Arsenic Variability

Building on the SIHA model (Hoque et al., 2017) —which stands for “Silt‐clay layers Impose Hierarchical groundwater flow patterns constraining Arsenic progression”—we explored how recharge zones, organic matter, and groundwater flow influence arsenic mobilization. The SIHA model integrates topographic elevation with subsurface lithological heterogeneity—particularly the presence of discontinuous silt‐clay layers—to simulate nested groundwater flow systems (local, intermediate, and regional). Within this framework, however, topography in deltaic systems is a relative construct. Even within basin‐scale topographic lows—such as the delta depocentre—local high points (e.g., natural levees, abandoned channels) can function as transient recharge zones, particularly during the onset of the monsoon. This dynamic means that over time and space, a single location can switch between being a recharge and discharge zone, governed by shifting hydraulic gradients, and its elevation relative to its immediate surroundings. Once recharging water enters the aquifer, its vertical movement is constrained by interleaved, discontinuous silt‐clay layers, while its lateral migration is influenced by surface topography in combination with these low‐permeability units. Together, these factors control the spatial retention of reduced groundwater and create geochemical conditions that promote release and accumulation of arsenic under anoxic environments.

Wallis et al. (2020) identified that arsenic hotspots arise from the co‐deposition of labile organic carbon and reactive iron oxides, coupled with advective fluxes that drive groundwater flow through these deposits. Arsenic release, however, only occurs when the flux of soluble electron acceptors (O_2_, NO_3_) is consumed rapidly enough by labile organic matter to create Fe‐reducing conditions (Bhattacharya et al., 1997; Ghosh et al., 2021; Glodowska et al., 2020; Lovley & Anderson, 2000; Nickson et al., 1998; Weber et al., 2006). These insights informed our approach to map recharge structures and assess their link to arsenic hotspots.

The Ayeyarwady Delta's alluvial terrain features nested catchments with hierarchical drainage networks (Vogel et al., 2024) (Figure S1 in Supporting Information S1). Recharge processes are diffuse, focused through micro‐depressions and ephemeral streams active after rainfall (Bixio et al., 2002; Cuthbert et al., 2013; Kmec & Šír, 2024). These depressions accumulate terrestrial organic matter, which decays and contributes carbon to the aquifer (Fellman et al., 2013). However, recharge and arsenic mobilization vary spatially, as reactive iron oxides and labile organic matter are not uniformly distributed (Meharg et al., 2006; Moore et al., 2024). High‐order streams primarily stay in equilibrium with adjacent groundwater, providing limited recharge (Ward, 1963). Focused recharge, therefore, is concentrated within the most numerous, lowest‐order ephemeral streams and landscape microdepressions, which together form a network of organic‐rich pathways.

The concentration of dissolved organic carbon (DOC) in shallow groundwater is dictated by recharge processes and influenced by biogeochemical and hydrological interactions (Datta et al., 2009; Ghosh et al., 2021; Harvey, Basu, et al., 2002; Hoque et al., 2017; Klump et al., 2006; Mailloux et al., 2013; Neumann et al., 2010; Stute et al., 2007; Yuan et al., 2025). Recharge and discharge zones often mimic the topographical hierarchy, with recharge in higher areas and discharge in lower areas. These zones facilitate water mixing and redox reactions, enhancing solute exchange. DOC often peaks at depth due to sluggish groundwater flow in anoxic conditions (Hoque et al., 2017). Consequently, local arsenic concentrations are shaped by recharge zones, organic matter dynamics, and interleaved clay‐silt layers that constrain flow within the aquifer.

Using SRTM 30 m DEM data, we applied the Hydrology Toolbox in ESRI ArcMap to delineate flow direction, accumulation, and stream order (Deilami & Hashim, 2011). Drainage density (Dd) was calculated as:

Dd=TotalStreamLengthCatchmentAreakmkm2

**Figure 1:**
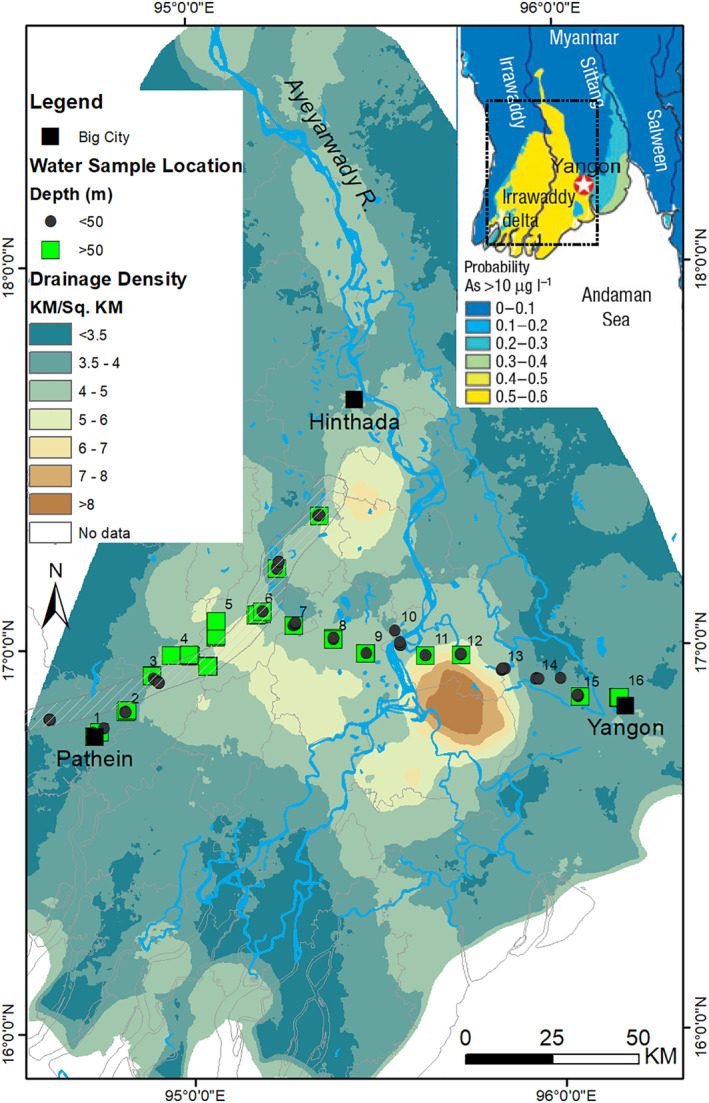
Distribution of sampling points relative to drainage density along a 170 km traverse across the Ayeyarwady Delta. This map illustrates a targeted study of potential arsenic‐contaminated aquifers along a transect from Pathein in the west to Yangon in the east, spanning varied drainage densities. We also included another transect with 55 measurements from an earlier study (van Geen et al., 2014) indicated in a hatched polygon. The inset map (Winkel et al., 2008) details the probability of exceeding a 10 μg/l arsenic concentration.

Using mapped drainage density data, we selected a traverse covering the variability in drainage density across a 170 km stretch of the Ayeyarwady Delta, from Pathein in the west to Yangon in the east (Figure 1). This route, aligned with accessible roads, allowed us to collect hydrogeochemical data at 16 locations. Sampling wells of varying depths captured arsenic's vertical variability, integrating spatial recharge structures with hydrogeochemical observations to inform predictive models.

### 3.2 Fieldwork, Water Chemistry and Isotopic Composition

Sampling was completed in September 2019 and each time GPS location and depth information were recorded. Eighty private wells (up to 215 m deep) were sampled, around 5 wells at each of 16 sites, covering the entire depth range of the available wells (Figure 1). These depths likely include Plio‐Pleistocene deposits; by comparison, Holocene deposits in the nearby Bengal Delta are typically limited to 90 m (Goodbred & Kuehl, 2000). However, this study focuses on water chemistry and arsenic distribution, not sedimentology. With well screen lengths of 1–3 m, samples collected are treated as representing a point sample. A standard, systematically‐applied sampling procedure was followed. Although the private wells were in regular use we purged for around three borehole volumes of water prior to collection of samples. In every case Electrical Conductivity (EC), redox potential (ORP) and pH were measured for each site. Samples for laboratory analysis were collected in 15 mL polythene tubes, one acidified in the field with 0.15 mL of 50% Analar® nitric acid, one unacidified.

The major cations (Na, Ca, Mg, K) plus Fe, Mn, Si, P, Sr, Ba, B, Cr, Co, Ni, Cu, As, Mo, Cd, Sb and Pb were determined with a SpectroBlue ICP‐OES at the University of Portsmouth that were calibrated across appropriate concentration ranges with commercially‐available multi‐element solutions. The precision and accuracy of the analyses were monitored with Certified Reference Materials (CRMs) and replicate determinations, which were estimated to be approximately 5%.

Isotope ratios of oxygen and hydrogen (δ^18^O, δ^2^H) were analyzed using a Picarro Cavity Ring‐Down Spectroscopy (CDRS) laser instrument at the University of East Anglia. Results are presented in the standard per mil notation, using Vienna Standard Mean Ocean Water (‰VSMOW) as the reference. The analysis involved taking six repeated measurements of 2.2 μl samples to mitigate memory effects. Consistency in the data was assured by repeat measurements of various standards, including Norwich Tap Water (NTW), Greenland Ice Sheet Precipitation (GISP), and the USGS67400 and USGS64444, achieving precisions of 0.16‰ for δ^18^O and 1.05‰ for δ^2^H.

The collected data on groundwater composition, and well details, including depth, GPS and location are given in Table S1 of Supporting Information S1.

### 3.3 Statistical Modeling

In assessing the probabilities of arsenic concentrations exceeding 10 and 50 μg/l thresholds in the Ayeyarwady Delta, logistic regression modeling within the generalized linear models (GLM) framework was applied (Ayotte et al., 2016; Winkel et al., 2008). This approach establishes a linear relationship from the provided data set and introduces nonlinearity through the Sigmoid function, encapsulating the relationship between the binary dependent variable—whether arsenic concentration exceeds a threshold—and independent variables.

The model expresses the logit—the natural logarithm of the odds ratio, which is the fraction of the probability of success (*P*) to the probability of failure (1−P) of an event's occurrence:

$$\text{logit}\left(P\right)=\ln\;\left(\frac{P}{1-P}\right)$$

The logit function, linked to the independent variables via linear regression, is then mapped back to the probability scale [0, 1] using the sigmoid function (Hosmer Jr et al., 2013):

$$P=\frac{1}{1+e^{-\left(\beta_{0}+\beta_{1}\times Sl+\beta_{2}\times D_{d}+\beta_{3}\times S_{n}\right)}}$$

This non‐linear transformation is essential since the binary response does not satisfy the ordinary least squares assumptions. Therefore, the maximum likelihood method is employed for coefficient estimation, assuming a Bernoulli distribution for the binary dependent variable (Agresti, 2015; Hosmer Jr et al., 2013). The Bernoulli distribution is a discrete probability distribution for a random variable that takes the value 1 with probability *P* and the value 0 with probability 1−*P*, suitable for modeling binary outcomes such as “success” or “failure”.

In the logistic regression model, *β*_0_ is the model's intercept, representing the log odds of the event occurring when all predictors are held at zero. The coefficients *β*_1_, *β*_2_, and *β*_3_ correspond to the variables median slope (Sl), mean drainage density (Dd), and median sand fraction in top 2 m (Sn), respectively, and quantify the change in the log odds of the arsenic concentration exceeding the threshold per unit change in each predictor variable. These variables were selected based on previous studies (e.g., Winkel et al., 2008) and their demonstrated relevance in predicting arsenic concentrations.

Variable selection was carefully considered, with a stepwise approach (stepAIC) employed to optimize the model by including and excluding predictors iteratively, guided by the Akaike Information Criterion (AIC). The final model selected is parsimonious yet effective in capturing the essential predictors (Table 1).

**Table 1:** Logistic Regression Model Coefficients for Predicting Arsenic Concentration Exceedance

Threshold (μg/l)	Variable	Coefficient (Estimate, *β*_0_, *β*_1_, *β*_2_, *β*_3_)	Standard Error	Wald statistic	p‐value
10	Intercept	1.7164	3.05200	0.562	0.573859
	Sl	−2.6733	0.76882	−3.477	0.000507
	Dd	1.1925	0.45093	2.644	0.008182
	Sn	−0.0864	0.06259	−1.381	0.167388
50	Intercept	0.3519	3.21567	0.109	0.91286
	Sl	−2.1915	0.76582	−2.862	0.00421
	Dd	0.7973	0.49176	1.621	0.10496
	Sn	−0.0385	0.06132	−0.628	0.52982

*Note:* The estimated coefficients from logistic regression models used to predict the probability of arsenic concentrations exceeding 10 and 50 μg/l thresholds. For each threshold, the table lists the model intercept and coefficients for median slope (Sl), mean drainage density (Dd), and median sand fraction in the top 2 m (Sn), along with their respective standard errors, Wald statistics, and p‐values. Standard errors reflect the variability and precision of the coefficient estimates. Wald statistics are used to test the hypothesis that a particular coefficient is zero, with larger values indicating a stronger relationship between the predictor and the outcome. P‐values provide the probability of observing such a Wald statistic if the null hypothesis (that the coefficient is zero) were true, with values less than 0.05 typically considered statistically significant.

A data set comprising 81 samples collected by our team, augmented with 55 samples from van Geen et al. (2014), was split into training (80%) and test (20%) sets for model fitting and validation. Model performance was demonstrated by the high accuracy rates—87.5% for the 10 μg/l and 75% for the 50 μg/l thresholds—indicative of the model's robust predictive power.

By applying the model coefficients to spatial raster data sets, we calculated the probability of arsenic concentration exceedance across the Delta. The transformed data delineate mean probability values for geographic blocks within administrative units, allowing us to pinpoint and visualize potential hotspots of arsenic contamination effectively.

## 4 Results

### 4.1 Drainage Density

Drainage density, which serves as an indicator of hydrological dynamics, was generally highest in the central delta, with some areas exhibiting particularly high concentrations of channels (Figure 1). The distribution of arsenic also demonstrates considerable variability. Spatial analysis reveals a non‐uniform but notable spatial association. The most elevated arsenic levels, exceeding 100 μg/l, coincide with regions of moderate drainage density. In contrast, arsenic concentrations between 10–50 μg/L and 50–100 μg/l are found across a range of drainage densities. Lower arsenic concentrations, generally below 10 μg/l, are predominant in areas of low drainage density. In addition to these general trends, the data set reveals significant heterogeneity, as exemplified by the co‐occurrence of high and low arsenic concentrations within areas of similar drainage density.

It is important to clarify that while drainage density may influence recharge processes and the movement of organic matter into shallow aquifers, groundwater flow within the aquifer is primarily controlled by topography and interleaved pattern of clay and silt layers, as detailed in Hoque et al. (2017). These subsurface heterogeneities dictate localized and vertically constrained recharge and discharge zones, typically limited to shallower depths (Tóth, 1963; Zijl, 1999). The observed association between drainage density and arsenic concentration likely reflects this interplay, with variability introduced by subsurface lithological differences.

### 4.2 Hydrochemical Variability Across the Ayeyarwady Delta

**Figure 2:**
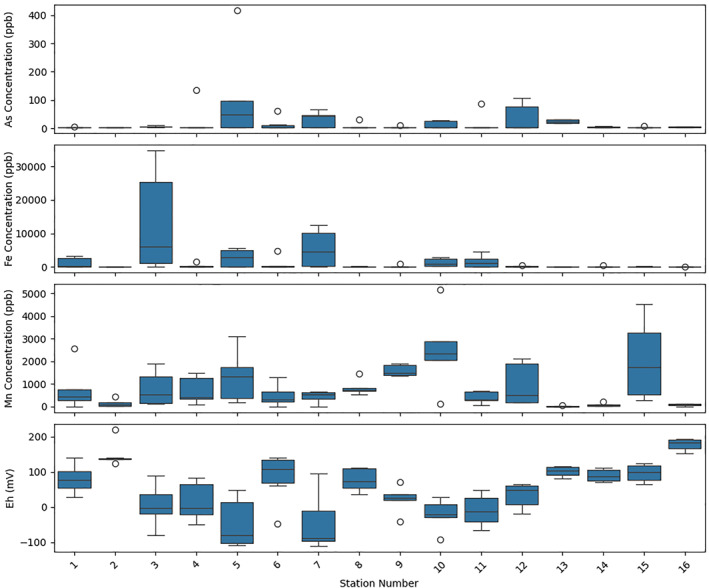
Hydrochemical profiles of groundwater samples across the Ayeyarwady Delta. Box plots represent the variability and median concentrations of arsenic (As), iron (Fe), manganese (Mn), and redox potential (Eh) measured at 16 stations along a west‐to‐east traverse of the delta. Each station reflects data from 5 to 6 groundwater samples. The plots illustrate the range of arsenic levels, some exceeding health guideline thresholds, and corresponding variations in Fe and Mn concentrations, with Eh values indicating the prevailing redox conditions. This composite view highlights the heterogeneity of groundwater quality and suggests redox control as a significant factor in arsenic mobilization within the delta's aquifer systems.

The distribution of arsenic and other key ions was investigated across a west‐to‐east traverse of the Ayeyarwady Delta, covering 16 sampling stations (Figure 2). Each station consisted of 5–6 groundwater samples, analyzed for variations in arsenic, iron, manganese, and redox potential (Eh). The box plots illustrate the range and median values across the stations. A noticeable spread in arsenic levels is evident, with several stations exhibiting median values above the World Health Organization's guideline of 10 μg/l, indicating potential health risks. Variability within stations, as indicated by the interquartile ranges, suggests heterogeneous arsenic distribution within localized groundwater sources.

Concomitant fluctuations in iron and manganese concentrations were observed, with some stations displaying high median values, particularly where arsenic concentrations are elevated. This trend may reflect the geochemical interactions within the aquifer system, where redox processes are known to influence the mobilization of these metals as well as arsenic.

Eh values are indicative of the redox state of groundwater, and varied widely across the stations. Several stations exhibited median Eh values within the reducing range, a state known to facilitate the mobilization of arsenic from sedimentary matrices into groundwater.

Collectively, the hydrochemical profiles assembled from this traverse suggest a complex interplay between geology, hydrology, and biogeochemical processes influencing aquifer chemistry, particularly arsenic levels, across the delta.

**Figure 3:**
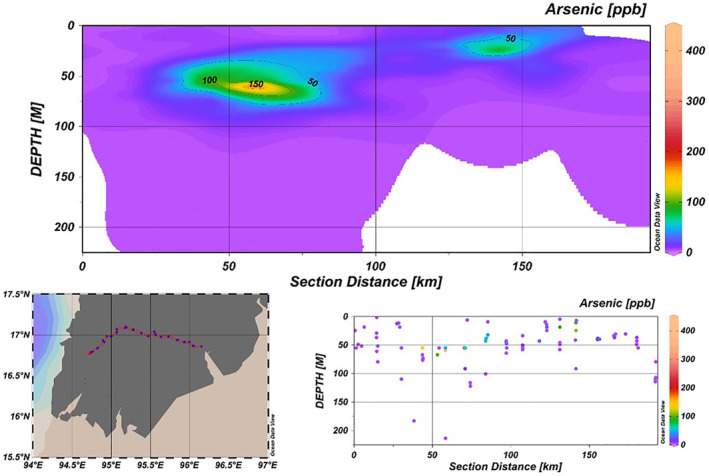
Depth‐profile and spatial distribution of arsenic concentrations along a transect in the Ayeyarwady Delta. The upper contour plot illustrates the variation of arsenic concentrations with depth, indicating regions within the aquifer where arsenic levels are significantly higher. The lower scatter plot and accompanying map provide a granular view of arsenic distribution across different depths and distances, with the dots colored according to arsenic concentration levels. These visualizations together offer insights into the vertical and horizontal heterogeneity of arsenic within the aquifer system.

Arsenic concentrations along a transect of the Ayeyarwady Delta were also analyzed to understand their distribution with respect to depth (Figure 3). The upper panel displays a contour plot indicating arsenic concentration gradients with depth, revealing zones of elevated arsenic levels that are not uniform across the transect. A notable peak in arsenic concentration, exceeding 150 μg/l, occurs at an intermediate depth around the 60 km mark, with a smaller, secondary zone of elevated arsenic near 150 km. This suggests the presence of distinct arsenic‐rich layers or pockets within the aquifer.

The lower panel complements the upper and presents discrete arsenic concentration data points along the same transect. This scatter plot highlights the depth‐specific variability of arsenic concentrations, primarily above 100 m, noting a scarcity of data points at greater depths.

### 4.3 Cation Chemistry and Electrical Conductivity

**Figure 4:**
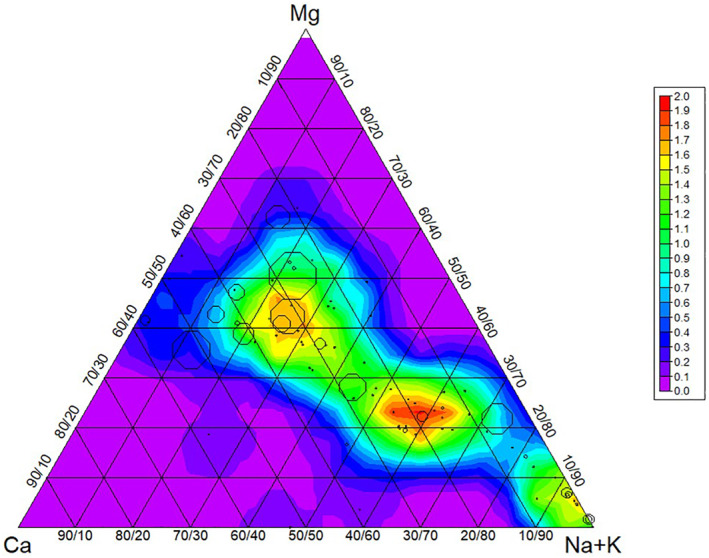
Trilinear plot summarizing the major cation composition across all groundwater samples. This plot illustrating the relative concentrations of sodium and potassium (Na + K), calcium (Ca), and magnesium (Mg) across the entire set of samples. While the majority of samples display a mixed cationic composition, zones of sodium dominance are present in some areas. Octagonal circles show arsenic concentrations, larger the radius higher the concentration.

**Figure 5:**
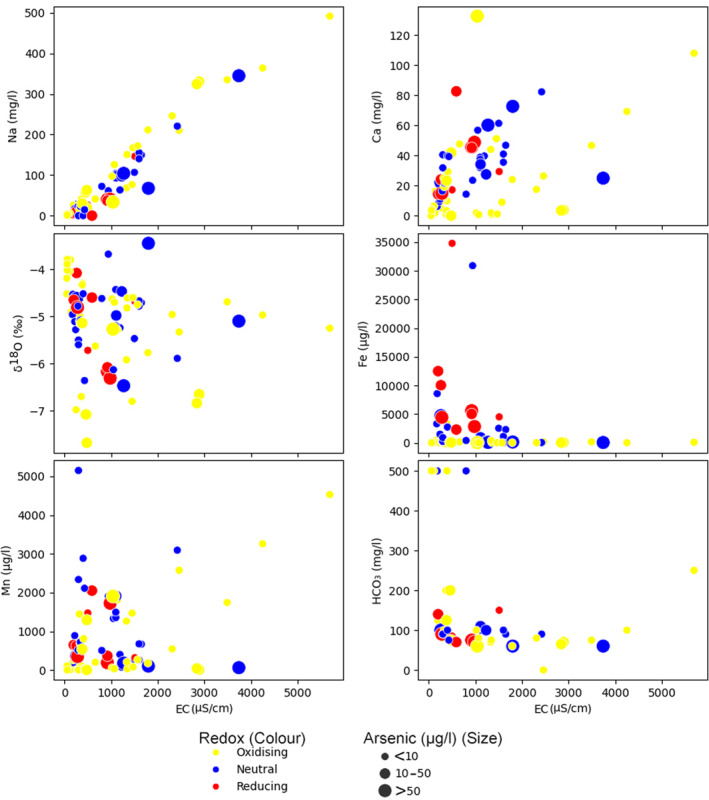
Multi‐parametric scatter plots displaying the interplay between EC and various hydrochemical constituents across different redox conditions and arsenic concentration categories.

The hydrochemical characterization is further explored with a trilinear plot representing the major cation composition (Na, K, Ca, Mg) of groundwater samples from the entire data set (Figure 4). The data spread across the plot reveal that the cationic composition of the groundwater is diverse, with no single type of cation predominating in the majority of samples and arsenic polluted wells have Ca‐Mg type water However, there are instances where sodium (Na) is notably prevalent, and those have higher EC (Figure 5).

Scatter plots (Figure 5) demonstrate a general increase in concentrations of sodium (Na) and calcium (Ca) with higher electrical conductivity (EC), suggesting a correlation with mineral solubility in the groundwater. Iron (Fe) and manganese (Mn) exhibit a wide range of concentrations, with higher levels often occurring under reducing conditions, as indicated by the red symbols. Reducing conditions are also associated with the larger symbol sizes, representing arsenic concentrations exceeding 50 ppb, consistent with a co‐mobilization process under reductive dissolution of oxides.

The bicarbonate (HCO_3_) plot against EC does not show a uniform trend, implying the influence of multiple geochemical processes. δ^18^O reveals a slight trend toward more negative values with increasing EC, possibly indicating evaporative enrichment in some groundwater samples.

The Eh categories (symbol color) reveals distinct redox groupings, with oxidising conditions often coinciding with lower concentrations of redox‐sensitive elements such as Fe and Mn. In contrast, reducing conditions align with greater variability in these element concentrations.

### 4.4 Co‐Occurrences of Iron and Manganese

**Figure 6:**
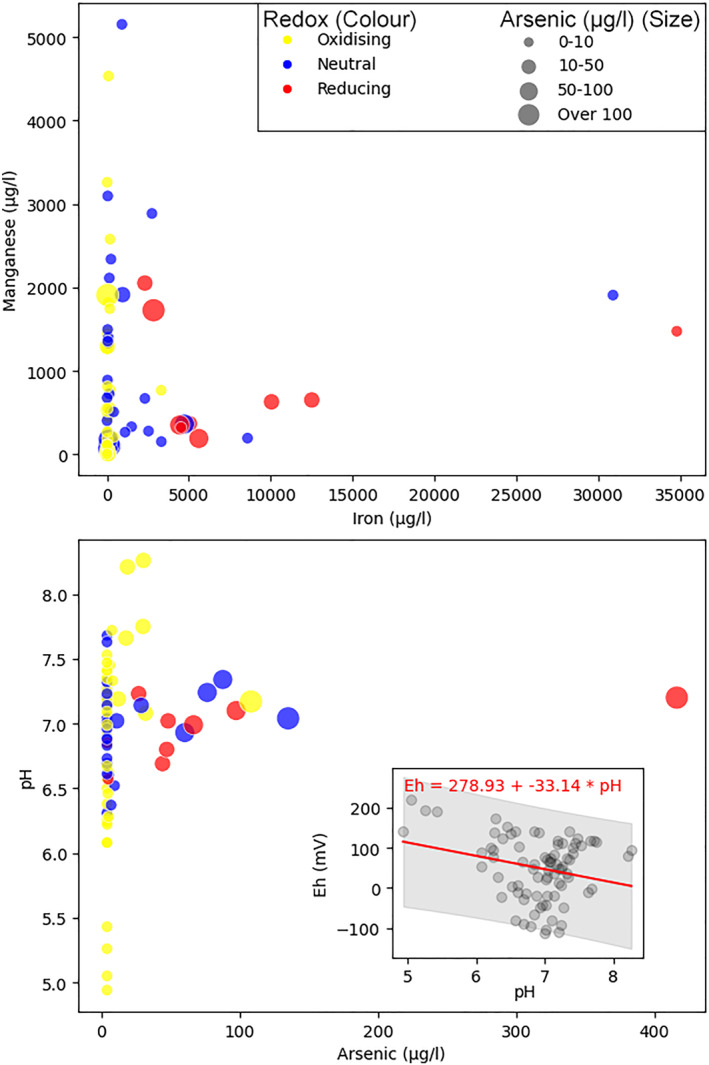
Bivariate plots of manganese versus iron concentration and arsenic versus pH, with redox state indicated by color and arsenic concentration by point size. The inset (bottom right) presents the relationship between pH and Eh, with the regression line (red) and 95% confidence interval (gray shaded area). The data indicate a higher arsenic presence at neutral pH levels, while lower pH correlates with below detection limit arsenic concentrations and oxic to neutral conditions. The significant scatter in the inset plot suggests that redox conditions are influenced by a variety of geochemical processes and constituents.

In the observed groundwater samples, iron concentrations up to approximately 35,000 μg/l and manganese up to 5,000 μg/l were recorded (Figure 6). The highest concentrations of manganese occur alongside substantial iron concentrations, suggesting a common geochemical or mineralogical source, such as the dissolution of manganese and iron‐bearing minerals. The arsenic concentrations over 50 μg/l, are predominantly associated with reducing conditions, consistent with geochemical expectations that arsenic release is enhanced in reducing environments due to the reductive dissolution of iron hydroxides. Oxidising conditions generally coincide with lower concentrations of manganese and iron probably due to oxidative precipitation.

The analysis of the aqueous geochemistry data suggests a correlation between pH and arsenic concentration within the aquifer system (Figure 6). Notably, arsenic concentrations tend to increase with pH, peaking at around neutral pH levels. Conversely, arsenic levels are consistently below detection thresholds in samples with lower pH, that are also more oxic to neutral redox conditions. The inset plot further elucidates the redox dynamics of the system. The substantial dispersion of data points around the regression line suggests that a single redox process is not solely responsible for the observed conditions. Instead, it appears that a suite of minerals, likely including iron and manganese oxyhydroxides, contribute to the redox heterogeneity (Frisbie et al., 2024). These observations underscore the complexity of the redox processes within the aquifer, indicating a system influenced by multiple concurrent geochemical reactions (Mukherjee et al., 2008).

### 4.5 Isotopic Composition

**Figure 7:**
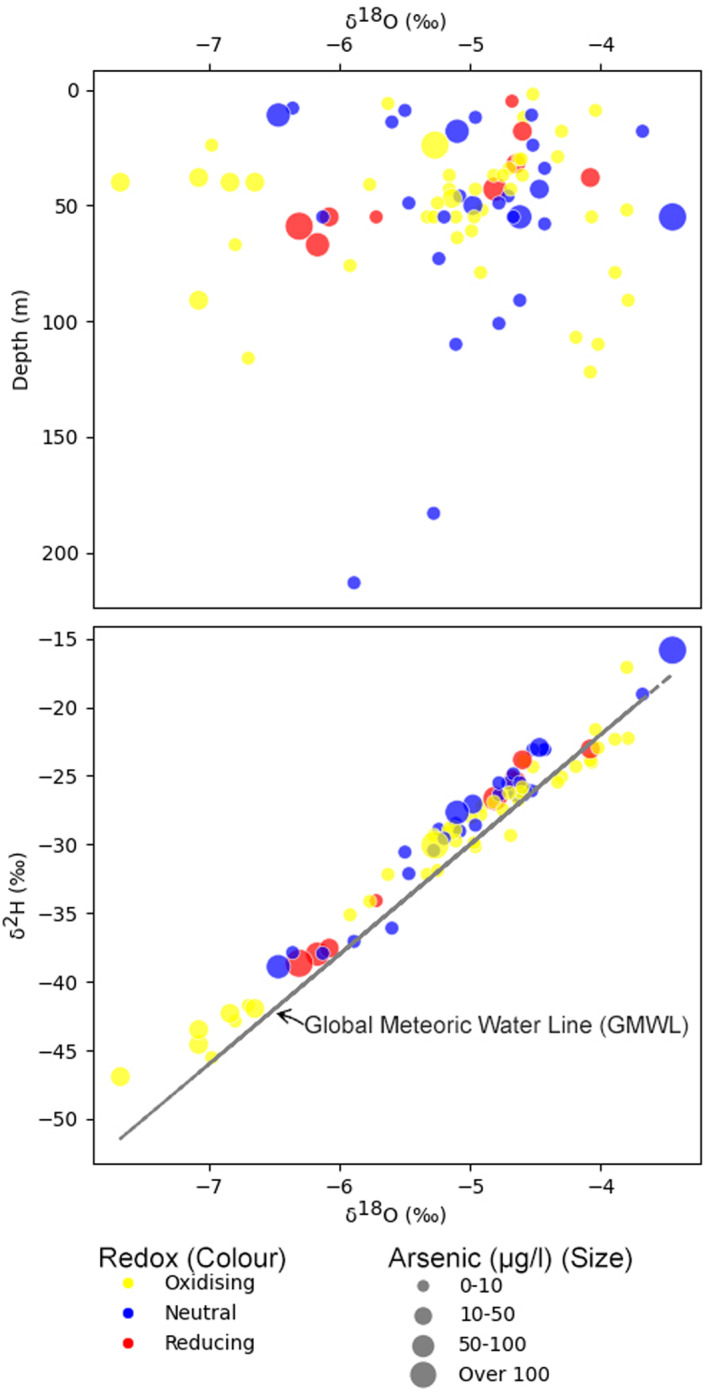
Isotopic analysis of groundwater samples from the Ayeyarwady Delta. Lower panel presents a cross‐plot of δ^18^O versus δ^2^H values in comparison to the Global Meteoric Water Line (GMWL), demonstrating the meteoric origin of groundwater recharge. Upper panel depicts the variation of δ^18^O with depth, revealing a wide range of values in the shallower aquifer zones and suggesting multiple groundwater bodies with minimal mixing. The lack of a clear vertical gradient indicates distinct recharge events across the rainy season I different zones which are hydraulically isolated rather than a homogenized system.

Isotopic analyses were conducted to constrain the sources and mixing of groundwater within the Ayeyarwady Delta. The lower plot (Figure 7) illustrates the δ^18^O and δ^2^H in relation to the global meteoric water line (GMWL). The proximity of the samples to the GMWL and the linear trendline through the data set suggest that the groundwater recharge is derived from rainfall (Clark & Fritz, 2013).

Conversely, δ^18^O plotted against depth (Figure 7, upper plot) displays a broad range, particularly in the shallower zones. This suggests minimal mixing between separate groundwater masses, and implies that recharge (through the rainy season) and groundwater movement occur in discrete pockets within the aquifer system, with limited hydrological connectivity at the examined depths.

### 4.6 Principal Component Analysis of Groundwater Chemistry

**Figure 8:**
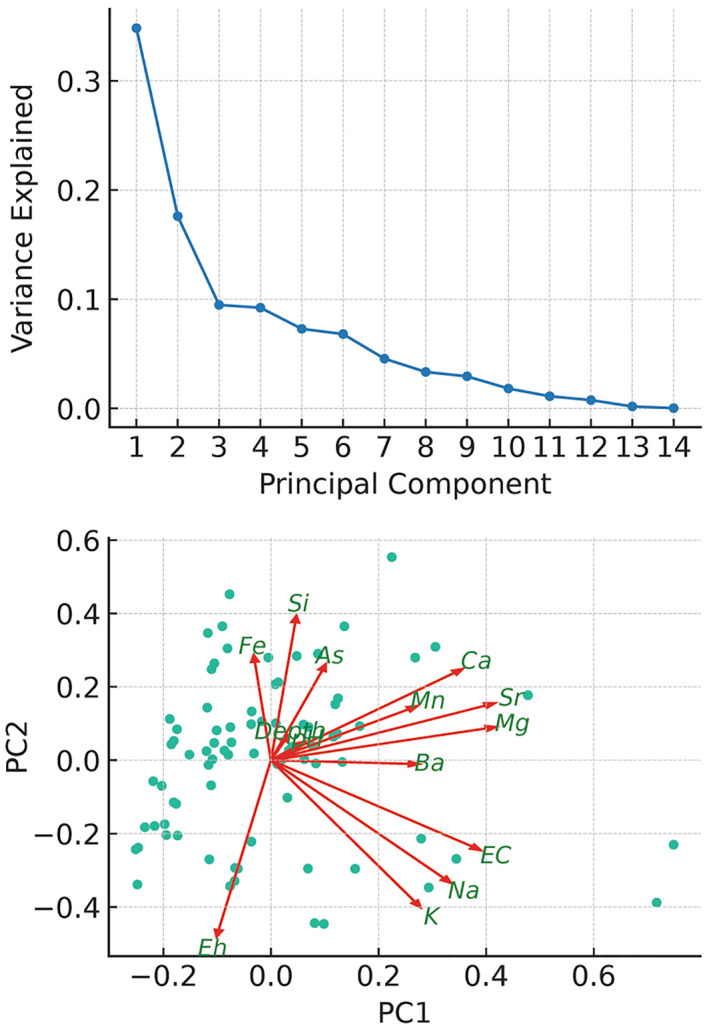
PCA of Groundwater Geochemistry in the Ayeyarwady Delta. Top panel displays the scree plot indicating the variance explained by each principal component, with PC1 to PC3 accounting for the majority of the variance. Bottom panel represents the loading plot for PC1 and PC2, with vectors indicating the direction and magnitude of each variable's contribution to the components. The superimposed scatter plot of sample scores suggests the geochemical clustering within the groundwater system.

Principal component analysis (PCA) was employed to discern the underlying hydrogeochemical processes influencing groundwater quality within the Ayeyarwady Delta (Figure 8). The first three components cumulatively explain 61.91% of the variance. The first principal component (PC1) accounted for 34.83%, with major contributions from magnesium (Mg), strontium (Sr) and electrical conductivity (EC), with sodium (Na) and calcium (Ca) to a lesser extent, indicative of the groundwater's general mineralization or salinity. The second principal component (PC2) was characterized by negative loadings from potassium (K) and positive loadings from silicon (Si), iron (Fe) and redox potential (Eh), possibly reflecting silicate weathering or redox variation.

Depth shows a significant positive loading on the third principal component (PC3), highlighting vertical geochemical stratification within the aquifer, including the distribution of arsenic. The influence of pH and Eh on PC3 alludes to the importance of acid‐base and redox conditions.

The consistent roles of Mg, Ca, EC, and Sr across the PCA suggest that indicators of mineralization and salinity are integral to comprehending the overall hydrogeochemistry of the groundwater in the delta.

### 4.7 Spatial and Statistical Modeling of Arsenic Concentrations

**Figure 9:**
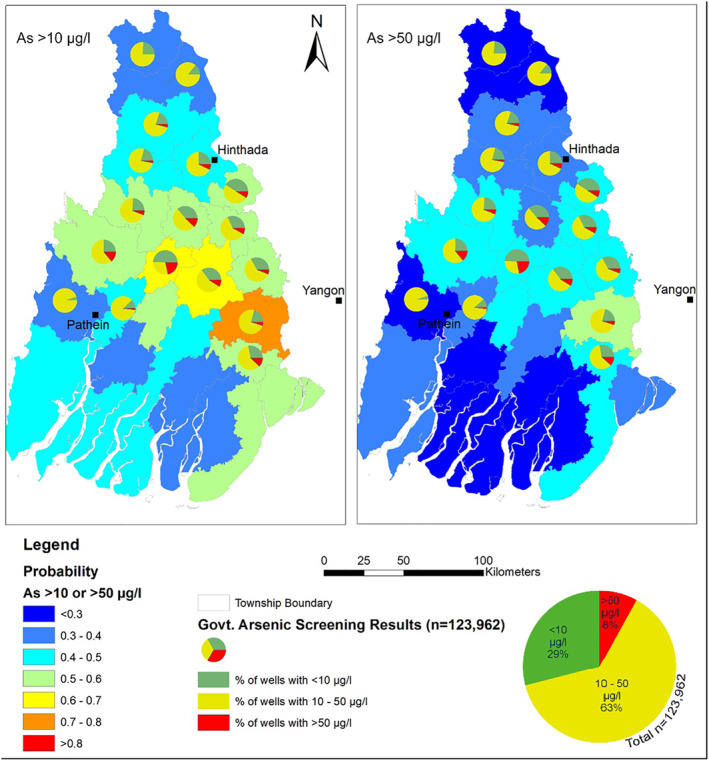
Spatial prediction of arsenic concentrations and comparative analysis with government data. Left Panel shows the mean predicted probability of groundwater arsenic concentrations exceeding the 10 μg/l threshold across the Ayeyarwady Delta. Right Panel represents predictions for the 50 μg/l threshold and both includes pie charts that overlay the predictions, indicating the proportion of tubewells above 10 μg/l and 50 μg/l, based on governmental data. These predictions validate the logistic regression model and highlight areas potentially impacted by arsenic contamination, aiding in targeted risk management and mitigation strategies.

After a stepwise selection process, logistic regression modeling identified median slope, mean drainage density, and median of sand in top 2 m as predictor of probability of arsenic concentrations exceeding both 10 and 50 μg/l thresholds. Spatial predictions from the model were mapped to demonstrate areas at elevated risk of surpassing arsenic thresholds of 10 and 50 μg/l (Figure 9).

The maps also featured pie charts based on approximately 125,000 tests that overlaid the predictions, incorporating both the fraction of tubewells exceeding 10 μg/l and those above 50 μg/l, thus providing a comprehensive view of the risks across the delta. For both thresholds, hotspot areas are concentrated in the mid‐central region of the delta.

## 5 Discussion

### 5.1 Spatial Variability of Arsenic is Prevalent in the Central, Shallower Aquifers of the Delta

The distribution of arsenic within the Ayeyarwady Delta exhibits notable spatial variability, a crucial factor in understanding and managing the potential health risks associated with groundwater consumption. Our investigation into the vertical profile of arsenic has demonstrated distinct layers within the aquifer system, with high concentrations restricted to shallower depths (<100 m), consistent with studies in similar fluvial settings (Fendorf et al., 2010; Ravenscroft et al., 2009; van Geen et al., 2014).

The variable distribution of arsenic across the delta relates to the complex interplay between geochemical processes and aquifer heterogeneities. Isotopic analysis (δ^18^O and δ^2^H) confirms that groundwater is primarily meteoric in origin. However, the wide range of δ^18^O values at shallower depths, combined with the absence of a clear verical gradient, implies that recharge is not a uniform process. Instead, groundwater at shallower depths exhibits signs of minimal mixing between separate water bodies (Figure 7). This indicates that recharge occurs in distinct, localized episodes, with recharge continuing throughout the monsoon in hydraulically segregated zones that remain above the floodwaters.

These dynamics are governed by transient hydrological functions that are strongly influenced by the hierarchical topography and seasonal water levels across the delta. In this multi‐scalar landscape, whether a specific location functions as a recharge or discharge zone is not fixed; it depends on its elevation relative to its immediate surroundings and the prevailing hydraulic head. This explains why recharge can be initiated in seemingly low‐lying areas, provided they are locally elevated above the current water table. The entire system is in a constant state of flux, with recharge zones shifting in response to even minor changes in water levels.

The effect of this is observable throughout the seasonal cycle. During peak flooding, recharge is limited to the highest elevation catchments, as the expansive low‐lying areas are saturated and function as discharge zones. However, this role reverses in the early stages of the rainy season. In this critical window, the regional water table is low, allowing these same organic‐rich lowlands to become active, focused recharge zones. This insight into a hierarchical framework helps to explain previous observations that linked flooding to high arsenic concentrations (Connolly et al., 2022) by demonstrating the specific, hydrodynamically compatible mechanism: recharge initiated on local high points (such as natural levees and hummocks) that sit above the waterline, providing a direct conduit for injecting surface‐derived organic carbon into the shallow aquifer.

Conversely, the larger rivers entrenched in the regional landscape function as natural discharge zones under baseflow conditions, exporting groundwater along the regional gradient. However, their corridors contain features like sub‐channels, shoals, and levee‐bound depressions that trap organic‐rich water during falling stages enabling short‐lived, spatially constrained recharge into adjacent aquifers. Yet, because these rivers occupy regional depressions with a downstream slope, any recharge entering the aquifer near their margins is unlikely to propagate laterally over large distances. Instead, the groundwater often re‐emerges into the river system further downstream. This mechanism of limited lateral connectivity, combined with organic matter accumulation and restricted flushing, helps explain the presence of localized arsenic hotspots near major riverbanks (Connolly et al., 2022; Covatti et al., 2023; Rahman et al., 2021; Stahl et al., 2016).

Our statistical modeling provides quantitative support for this conceptual framework. The key predictive variables we identified—median slope, drainage density, and median sand content—are all physical expressions of the transient hydrological functions and hierarchical topography previously discussed. These factors underpin the topography‐driven recharge mechanisms that control the transport and distribution of arsenic. Our findings corroborate the critical roles that landscape elevation and nested hydrological regime play in arsenic distribution, aligning with observations from similar fluvial environments (Amini et al., 2008; Winkel et al., 2008). This holistic approach enhances our understanding of the interplay between the variable topography of the delta and the spatial patterning of arsenic contamination, setting a foundation for targeted mitigation strategies.

### 5.2 Predictive Models Show Arsenic Hotspots in the Deltas Mid‐Central Region

Our statistical models reveal the probabilistic distribution of arsenic across the Ayeyarwady Delta, with the models employing median slope and drainage density as reliable indicators. Median slope shows a negative correlation, while drainage density exhibits a positive correlation with arsenic presence. This dual‐factor analysis offers a robust method for identifying potential hotspots within the delta, as depicted in Figure 9.

Areas with highest probabilities of exceeding the 10 μg/l arsenic are concentrated in the mid‐central regions south of Hinthada and near Yangon. These regions are characterized by higher drainage densities, which, in the delta's hierarchical catchments, correspond to lower‐order streams that drain local topographic highs. As established in the previous section, this hierarchical fractal structure means that even regional lowlands act as depocenters for dissolved organic matter (DOM) while still facilitating focused recharge through these lower‐order streams (Fellman et al., 2013; Sieczko & Peduzzi, 2014; Wen et al., 2020). These process delivers organic matter into the shallow subsurface during recharge events, creating conditions that promote arsenic mobilization in the shallow subsurface (Hoque et al., 2017; Moore et al., 2024). Thus the observed spatial association between surface recharge processes and arsenic hotspots reflects the interplay of topography, transient recharge, and subsurface heterogeneity.

The arsenic hotspots in the delta's mid‐central regions are consistent with patterns observed in the lower and middle parts of other major south and southeast Asian deltas (Connolly et al., 2022; Shamsudduha et al., 2009; Sieczko & Peduzzi, 2014) and along major rivers (Chakraborti et al., 2013; Rahman et al., 2021). The relatively high organic carbon concentrations in these areas can be attributed to a low hydraulic gradient, warmer temperatures, and extensive agricultural and residential land use, which collectively enhance organic matter availability and its transport into aquifers.

The predictive reliability of our model is validated by government screening results (Phyu, 2019), which show a higher percentage of wells with arsenic levels above 10 and 50 μg/l in these same areas. However, we acknowledge that our statistical approach integrates samples across depth intervals, which may introduce variability due to subsurface heterogeneities. The probabilistic predictions are not intended to resolve detailed aquifer stratigraphy but rather to identify high‐risk zones for targeted screening and management efforts. This spatial alignment of model predictions with empirical data underlines the applicability of our approach for practical groundwater management, particularly in the absence of detailed sedimentological data.

### 5.3 Overlapping Redox and Differential Flushing Control Arsenic Levels

As established, organic matter that accumulates and decomposes in low‐lying areas serves as the fuel for arsenic mobilization when these locations function as a recharge zones for a limited time each year (Meharg et al., 2006; Shamsudduha et al., 2009). The introduced dissolved organic matter strongly influences redox reactions, driving the removal of oxidants and maintaining reducing conditions that keep arsenic in solution (Hoque et al., 2017; Wallis et al., 2020). This explains why low‐lying areas and regions near large rivers are arsenic hotspots (Fendorf et al., 2010; Rahman et al., 2021), a pattern consistent with observations in the Ayeyarwady Delta.

Building on the SIHA framework, our findings suggest that while subsurface heterogeneity controls aquifer's internal distribution and flushing of arsenic, the transient recharge processes discussed above are what fuel the system by controlling the delivery of organic matter. As expected, one primary control on arsenic mobility is redox conditions, indicated by the significant loading of redox potential (Eh) on the principal components (Figure 8). The reduction of iron oxides under anaerobic conditions—a process well documented in other deltas (Berg et al., 2001; BGS/DPHE, 2001; Bhattacharya et al., 1997; Fendorf et al., 2010; Nickson et al., 1998)—plays a pivotal role. This is corroborated by the positive correlation between iron concentration and arsenic levels in groundwater (Figure 2).

The geochemical signature is characterized by high magnesium (Mg), strontium (Sr) and electrical conductivity (EC). Given likely meteoric water recharge, the presence of these ions at significant concentrations suggests mineral dissolution influences arsenic behavior (Frisbie et al., 2024).

The link between our conceptual model and statistical findings is further reinforced by the predictor variables. Drainage density, as a proxy for the hierarchical recharge pathways, has emerged as a positive factor in arsenic presence. Conversely, the negative association with median slope reflects the impact of topography on groundwater flow and flushing.

The Ayeyarwady Delta exhibits arsenic concentrations that are relatively low compared to other deltas within Southeast Asia, such as the Ganges‐Brahmaputra and Mekong deltas. This distinction can be attributed to a variety of geological, hydrological, and biogeochemical factors, such as the area's unique sedimentary and hydrological characteristics, which may facilitate more effective flushing of arsenic from the aquifer system (Hoque et al., 2014, 2017; McArthur et al., 2011; van Geen et al., 2008). Faster groundwater flow rates could lead to lower arsenic concentrations, a hypothesis that warrants further investigation in the context of the Ayeyarwady Delta (van Geen et al., 2014).

The sedimentary environment of the Ayeyarwady Delta may also contribute to this variation. The delta's sediment composition, sourced from the eastern Indo‐Burman (Himalayan) orogeny, is likely distinct from that of other deltas leading to coarser sediments, which can influence arsenic release and flushing (Chen et al., 2020; Garzanti et al., 2016; van Geen et al., 2008). Further research is required to elucidate the precise mineralogical and geochemical characteristics of these sediments and their role in arsenic binding and release mechanisms. Additionally, the redox regime of the Delta may be less conducive to widespread arsenic mobilization, possibly due to more limited anaerobic degradation of organic matter or differences in microbial activity relative to other deltas.

**Figure 10:**
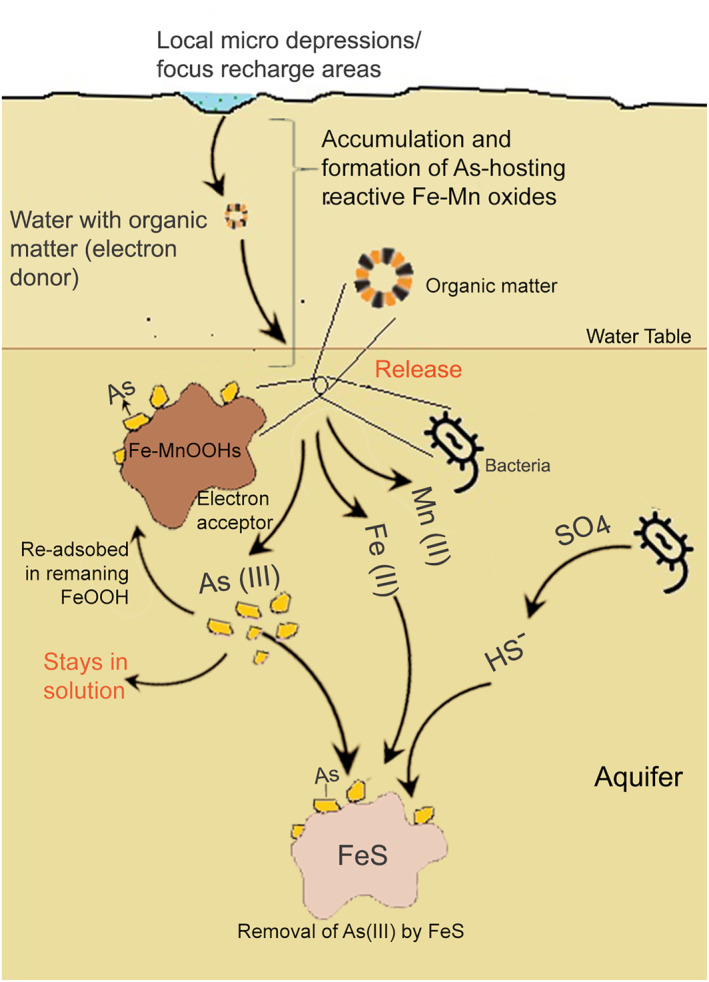
A conceptual diagram illustrating the mechanisms behind the low arsenic levels in the Ayeyarwady Delta's groundwater. Predominantly sandy sediments promote oxygenated water recharge and expedite arsenic flushing. In certain groundwater pockets, this process may lead to overlapping redox conditions, triggering the release of arsenic through MnOOH reduction or the partial reduction of FeOOH, which then allows arsenic to be adsorbed onto remaining FeOOH. Additionally, SO_4_ reduction in some areas could lead to the formation of HS^−^, which can precipitate Fe(II) as FeS, further removing As(III) from the solution. These processes, while not universally present, may operate in isolation or concurrently within some groundwater pockets, typically within the top 100 m of the delta.

Moreover, the vertical stratification implied by depth's dominance in the PCA analysis points to distinct aquifer layers with varying geochemical properties. The specific hydrogeochemical processes at different depths—such as competitive ion exchange, adsorption‐desorption reactions, and microbial activities—require detailed examination to elucidate their roles in arsenic mobilization. The overarching hydrochemistry may be controlled by overlapping redox as has been reported elsewhere in the region (Mukherjee et al., 2008) and release and sequestration of arsenic as we conceptualize in Figure 10. In particular, it is plausible that within parts of the aquifer, MnOOH is reduced before FeOOH, and that arsenic released during partial FeOOH reduction is subsequently sequestered onto remaining iron oxides—limiting its mobility (McArthur et al., 2004, 2008).

Furthermore, in the Ayeyarwady Delta, surface lithologies, primarily coarser, contribute to the formation of predominantly unconfined to semi‐confined, shallow aquifers. Examination of the arsenic profile (Figure 3) reveals that wells are generally situated at shallower depths, with the exception of regions approximately 50 km away from the western end, suggesting a potential scarcity of shallow aquifers due to the presence of finer materials.

It has been observed that arsenic concentrations are consistently higher in zones where a thick clay layer is present at shallow depths (van Geen et al., 2006). In the Bengal basin, low arsenic concentrations have been associated with rapidly recharged sandy aquifers that typically extend to the surface (Aziz et al., 2008; Hoque et al., 2009), a condition that may be analogous to those observed in the Ayeyarwady Delta. Although sulfate was not measured in our samples, a previous study (van Geen et al., 2014) indicated the presence of detectable levels of sulfate in many low‐arsenic wells in Ayeyarwady Delta, suggesting that the attenuation of arsenic might be facilitated by the precipitation of iron sulfides, as documented in Bangladesh and Vietnam (Buschmann & Berg, 2009; Lowers et al., 2007; van Geen et al., 2014). We also see a strong pattern with pH and higher arsenic concentration, and note that lower pH is often associated with lower or undetectable arsenic concentrations, potentially due to pH‐dependent removal of arsenic from solution (Han et al., 2011).

### 5.4 Re‐Evaluation of the Regulatory Standards for Arsenic in Groundwater is Needed

The relatively low arsenic levels are particularly significant considering the current regulatory thresholds for arsenic in groundwater across different Asian countries. Unlike India, which has adopted the World Health Organization's guideline of 10 μg/l, many countries, including Myanmar, continue to use the older thresholdof 50 μg/l.

Our study highlights the need for policymakers to re‐evaluate the existing regulatory standards. While only 8% of wells in the national screening data (Phyu, 2019) exceed the regulatory limit of 50 μg/l of Myanmar, a substantial 71% would be considered unsafe by WHO standards. This discrepancy has serious public health implications, particularly as Myanmar's “safe water for all by 2030” initiative increasingly promotes the use of groundwater. Without revising the regulatory threshold to align with WHO recommendations, the program risks widespread exposure to potentially harmful levels of arsenic.

The persistence of higher national standards may offer administrative convenience by designating more sources as “safe.” However, as highlighted by Meharg and Raab (2010) and Sharma (2017), the effectiveness of such standards is limited by weak enforcement and a lack of statutory authority. This creates a risk of what Nwankwo et al. (2020) term “political exposure”—where communities continue using water sources that meet national but not international safety thresholds.

Our findings make a compelling case for regulatory reform. Aligning arsenic standards of Myanmar with WHO guidelines would strengthen the scientific basis of water safety policy and reduce population‐level exposure risks. This shift is essential if national efforts to achieve universal access to safe drinking water by 2030 are to be both technically sound and protective of public health.

## 6 Conclusions

This study combined fieldwork, laboratory measurements, and predictive modeling to examine arsenic distribution across the Ayeyarwady Delta. Utilizing 81 samples collected along a 170 km transect from west to east, which was strategically aligned with hydrological features identified via digital elevation modeling, we identified arsenic hotspots primarily in the mid‐central regions where drainage density is higher. In comparison with other Asian deltas, the Ayeyarwady Delta exhibits lower frequencies of high‐arsenic wells, with 21% of our data set exceeding 50 μg/l regulatory limit held by Myanmar. However, nationally, only 8% of wells surpass this threshold, while a concerning 71% (*n* = 123,962) do not meet the 10 μg/l threshold by WHO, indicating persistent risks.

The study revealed that the delta's transient recharge patterns, governed by its hierarchical topography and the deltaic sedimentary environment, critically affect arsenic mobility and distribution. High arsenic levels are notably prevalent in areas with lower elevations and dense drainage networks, likely due to the co‐occurrence of organic matter and iron oxyhydroxides containing arsenic. Conversely, a negative correlation with median slope suggests that arsenic flushing occurs more effectively in areas with steeper topographical gradients.

Our findings highlight the urgent need to re‐evaluate existing arsenic regulatory standards. Despite relatively low arsenic levels compared to other Southeast Asian deltas, the substantial proportion of wells exceeding WHO's recommended 10 μg/l limit poses ongoing public health risks. Aligning the regulatory standards of Myanmar with WHO guidelines is imperative as the country strives to provide safe water by 2030, a goal that will likely depend heavily on groundwater resources.

Additionally, we advocate for the implementation of a comprehensive blanket‐screening program in identified hotspot areas. This proactive approach is essential for effectively managing arsenic contamination and mitigating its health impacts on the local population, ensuring the success of national initiatives aimed at safe water accessibility.

## Conflict of Interest

The authors declare no conflicts of interest relevant to this study.

## Supporting information

Supporting Information S1

## Data Availability

The data set supporting the findings of this study is publicly available through the University of Portsmouth research repository: Hoque. (2025). It includes groundwater composition, well details (depth, GPS coordinates, and location), and all parameters used in the analysis. Data are provided in spreadsheet format with a comprehensive README file. No proprietary or unpublished data were used in this study. All cited references are publicly accessible.

## References

[gh270063-bib-0001] ADB . (2013). Myanmar: Urban development and water sector assessment, strategy, and road map (Publication Stock No. RPT135826). Asian Development Bank (ADB). Retrieved from https://www.adb.org/documents/myanmar‐urban‐development‐and‐water‐sector‐assessment‐strategy‐and‐road‐map

[gh270063-bib-0002] Agresti, A. (2015). Foundations of linear and generalized linear models. John Wiley and Sons.

[gh270063-bib-0003] Amini, M. , Abbaspour, K. C. , Berg, M. , Winkel, L. , Hug, S. J. , Hoehn, E. , et al. (2008). Statistical modeling of global geogenic arsenic contamination in groundwater. Environmental Science and Technology, 42(10), 3669–3675. 10.1021/es702859e 18546706

[gh270063-bib-0004] Ayotte, J. D. , Nolan, B. T. , & Gronberg, J. A. (2016). Predicting arsenic in drinking water Wells of the Central Valley, California. Environmental Science and Technology, 50(14), 7555–7563. 10.1021/acs.est.6b01914 27399813

[gh270063-bib-0005] Aziz, Z. , van Geen, A. , Stute, M. , Versteeg, R. , Horneman, A. , Zheng, Y. , et al. (2008). Impact of local recharge on arsenic concentrations in shallow aquifers inferred from the electromagnetic conductivity of soils in Araihazar, Bangladesh. Water Resources Research, 44(7), W07416. 10.1029/2007WR006000

[gh270063-bib-0006] Bacquart, T. , Frisbie, S. , Mitchell, E. , Grigg, L. , Cole, C. , Small, C. , & Sarkar, B. (2015). Multiple inorganic toxic substances contaminating the groundwater of Myingyan Township, Myanmar: Arsenic, manganese, fluoride, iron, and uranium. Science of the Total Environment, 517, 232–245. 10.1016/j.scitotenv.2015.02.038 25748724

[gh270063-bib-0007] Berg, M. , Tran, H. C. , Nguyen, T. C. , Pham, H. V. , Schertenleib, R. , & Giger, W. (2001). Arsenic contamination of groundwater and drinking water in Vietnam: A human health threat. Environmental Science and Technology, 35(13), 2621–2626. 10.1021/es010027y 11452583

[gh270063-bib-0008] BGS/DPHE . (2001). Arsenic contamination of groundwater in Bangladesh (WC/00/19). (BGS Technical Report WC/00/19, Issue. Retrieved from https://www2.bgs.ac.uk/groundwater/downloads/bangladesh/reports/Vol2MainBook.pdf

[gh270063-bib-0009] Bhattacharya, P. , Chatterjee, D. , & Jacks, G. (1997). Occurrence of arsenic‐contaminated groundwater in alluvial aquifers from the Delta Plains, eastern India: Options for safe drinking water supply. Water Resources Development, 13(1), 79–92. 10.1080/07900629749944

[gh270063-bib-0010] Bixio, A. , Gambolati, G. , Paniconi, C. , Putti, M. , Shestopalov, V. , Bublias, V. , et al. (2002). Modeling groundwater‐surface water interactions including effects of morphogenetic depressions in the Chernobyl exclusion zone. Environmental Geology, 42(2), 162–177. 10.1007/s00254-001-0486-7

[gh270063-bib-0011] Buschmann, J. , & Berg, M. (2009). Impact of sulfate reduction on the Scale of arsenic contamination in groundwater of the Mekong, Bengal and Red River deltas. Applied Geochemistry, 24(7), 1278–1286. 10.1016/j.apgeochem.2009.04.002

[gh270063-bib-0012] Chakraborti, D. , Rahman, M. M. , Das, B. , Nayak, B. , Pal, A. , Sengupta, M. K. , et al. (2013). Groundwater arsenic contamination in Ganga–Meghna–Brahmaputra plain, its health effects and an approach for mitigation. Environmental Earth Sciences, 70(5), 1993–2008. 10.1007/s12665-013-2699-y

[gh270063-bib-0013] Chen, D. , Li, X. , Saito, Y. , Liu, J. P. , Duan, Y. , Liu, S. a. , & Zhang, L. (2020). Recent evolution of the Irrawaddy (Ayeyarwady) Delta and the impacts of anthropogenic activities: A review and remote sensing survey. Geomorphology, 365, 107231. 10.1016/j.geomorph.2020.107231

[gh270063-bib-0014] Clark, I. D. , & Fritz, P. (2013). Environmental isotopes in hydrogeology. CRC Press.

[gh270063-bib-0015] Connolly, C. T. , Stahl, M. O. , DeYoung, B. A. , & Bostick, B. C. (2022). Surface flooding as a key driver of groundwater arsenic contamination in Southeast Asia. Environmental Science and Technology, 56(2), 928–937. 10.1021/acs.est.1c05955 34951307 PMC8766940

[gh270063-bib-0016] Covatti, G. , Hoang, T. N. A. , & Grischek, T. (2023). Release of arsenic during riverbank filtration under anoxic conditions linked to grain size of riverbed sediments. Science of the Total Environment, 900, 165858. 10.1016/j.scitotenv.2023.165858 37516192

[gh270063-bib-0017] Cuthbert, M. , Mackay, R. , & Nimmo, J. (2013). Linking soil moisture balance and source‐responsive models to estimate diffuse and preferential components of groundwater recharge. Hydrology and Earth System Sciences, 17(3), 1003–1019. 10.5194/hess-17-1003-2013

[gh270063-bib-0018] Datta, S. , Mailloux, B. , Hoque, M. A. , Jung, H. B. , Stute, M. , Ahmed, K. M. , & Zheng, Y. (2009). Enrichment of arsenic in sediments from the Meghna river bank in Bangladesh: Implication for recycling of arsenic. PNAS, 106(40), 16930–16935. 10.1073/pnas.0908168106 19805180 PMC2761342

[gh270063-bib-0019] Deilami, K. , & Hashim, M. (2011). Very high resolution optical satellites for DEM generation: A review. European Journal of Scientific Research, 49(4), 542–554.

[gh270063-bib-0020] Donselaar, M. E. , Bhatt, A. G. , & Ghosh, A. K. (2017). On the relation between Fluvio‐deltaic flood basin geomorphology and the wide‐spread occurrence of arsenic pollution in shallow aquifers. Science of the Total Environment, 574, 901–913. 10.1016/j.scitotenv.2016.09.074 27665450

[gh270063-bib-0021] Fan, R. , Deng, Y. , Du, Y. , & Xie, X. (2024). Predicting geogenic groundwater arsenic contamination risk in floodplains using interpretable machine‐learning model. Environmental Pollution, 340, 122787. 10.1016/j.envpol.2023.122787 37879555

[gh270063-bib-0022] Fellman, J. B. , Petrone, K. C. , & Grierson, P. F. (2013). Leaf litter age, chemical quality, and photodegradation control the fate of leachate dissolved organic matter in a dryland river. Journal of Arid Environments, 89, 30–37. 10.1016/j.jaridenv.2012.10.011

[gh270063-bib-0023] Fendorf, S. , Michael, H. A. , & van Geen, A. (2010). Spatial and temporal variations of groundwater arsenic in south and Southeast Asia. Science, 328(5982), 1123–1127. 10.1126/science.1172974 20508123

[gh270063-bib-0024] Frisbie, S. H. , Mitchell, E. J. , & Molla, A. R. (2024). Sea level rise from climate change is expected to increase the release of arsenic into Bangladesh's drinking well water by reduction and by the salt effect. PLoS One, 19(1), e0295172. 10.1371/journal.pone.0295172 38232061 PMC10793926

[gh270063-bib-0025] Garzanti, E. , Wang, J.‐G. , Vezzoli, G. , & Limonta, M. (2016). Tracing provenance and sediment fluxes in the Irrawaddy River basin (Myanmar). Chemical Geology, 440, 73–90. 10.1016/j.chemgeo.2016.06.010

[gh270063-bib-0026] Ghosh, D. , & Donselaar, M. E. (2023). Predictive geospatial model for arsenic accumulation in Holocene aquifers based on interactions of oxbow‐lake biogeochemistry and alluvial geomorphology. Science of the Total Environment, 856, 158952. 10.1016/j.scitotenv.2022.158952 36150597

[gh270063-bib-0027] Ghosh, D. , Kumar, S. , Donselaar, M. E. , Corroto, C. , & Ghosh, A. K. (2021). Organic Carbon transport model of abandoned river channels—A motif for floodplain geomorphology influencing biogeochemical swaying of arsenic. Science of the Total Environment, 762, 144400. 10.1016/j.scitotenv.2020.144400 33385790

[gh270063-bib-0028] Giosan, L. , Naing, T. , Min Tun, M. , Clift, P. D. , Filip, F. , Constantinescu, S. , et al. (2018). On the Holocene evolution of the Ayeyawady megadelta. Earth Surface Dynamics, 6(2), 451–466. 10.5194/esurf-6-451-2018

[gh270063-bib-0029] Glodowska, M. , Stopelli, E. , Schneider, M. , Lightfoot, A. , Rathi, B. , Straub, D. , et al. (2020). Role of in situ natural organic matter in mobilizing as during microbial reduction of FeIII‐Mineral‐Bearing aquifer sediments from Hanoi (Vietnam). Environmental Science and Technology, 54(7), 4149–4159. 10.1021/acs.est.9b07183 32157881

[gh270063-bib-0030] Goodbred, S. L., Jr. , & Kuehl, S. A. (2000). The significance of large sediment supply, active tectonism, and Eustasy on margin sequence development: Late Quaternary stratigraphy and evolution of the Ganges‐Brahmaputra delta. Sedimentary Geology, 133(3–4), 227–248. 10.1016/s0037-0738(00)00041-5

[gh270063-bib-0031] Han, Y.‐S. , Gallegos, T. J. , Demond, A. H. , & Hayes, K. F. (2011). FeS‐coated sand for removal of arsenic(III) under anaerobic conditions in permeable reactive barriers. Water Research, 45(2), 593–604. 10.1016/j.watres.2010.09.033 20974481

[gh270063-bib-0032] Harvey, C. F. , Basu, A. R. , Jacobsen, S. B. , Poreda, R. J. , Dowling, C. B. , & Aggarwal, P. K. (2002). Groundwater flow in the Ganges Delta. Science, 296(5573), 1563a. 10.1126/science.296.5573.1563a 12040147

[gh270063-bib-0033] Harvey, C. F. , Swartz, C. H. , Badruzzaman, A. B. M. , Keon‐Blute, N. , Yu, W. , Ali, M. A. , et al. (2002). Arsenic mobility and groundwater extraction in Bangladesh. Science, 298(5598), 1602–1606. 10.1126/science.1076978 12446905

[gh270063-bib-0034] Hoque, M. (2025). Groundwater arsenic, depth, and location data from the Ayeyarwady Delta, Myanmar [Dataset]. University of Portsmouth. 10.17029/a0e9dc8b-3aa1-4e4e-82ae-578bc009e7b7

[gh270063-bib-0035] Hoque, M. A. , Burgess, W. G. , & Ahmed, K. M. (2017). Integration of aquifer geology, groundwater flow and arsenic distribution in deltaic aquifers—A unifying concept. Hydrological Processes, 31(11), 2095–2109. 10.1002/hyp.11181

[gh270063-bib-0036] Hoque, M. A. , Burgess, W. G. , Shamsudduha, M. , & Ahmed, K. M. (2011). Delineating low‐arsenic groundwater environments in the Bengal Aquifer System, Bangladesh. Applied Geochemistry, 26(4), 614–623. 10.1016/j.apgeochem.2011.01.018

[gh270063-bib-0037] Hoque, M. A. , Khan, A. A. , Shamsudduha, M. , Hossain, M. S. , Islam, T. , & Chowdhury, T. H. (2009). Near surface lithology and spatial variation of arsenic concentration in the shallow groundwater of Bangladesh. Environmental Geology, 56(8), 1687–1695. 10.1007/s00254-008-1267-3

[gh270063-bib-0038] Hoque, M. A. , McArthur, J. M. , & Sikdar, P. K. (2014). Sources of low‐arsenic groundwater in the Bengal Basin: Investigating the influence of the last glacial maximum palaeosol using a 115‐km traverse across Bangladesh. Hydrogeology Journal, 22(7), 1535–1547. 10.1007/s10040-014-1139-8

[gh270063-bib-0039] Hoque, M. A. , Scheelbeek, P. F. D. , Vineis, P. , Khan, A. E. , Ahmed, K. M. , & Butler, A. P. (2016). Drinking water vulnerability to climate change and alternatives for adaptation in coastal South and South East Asia. Climatic Change, 136(2), 247–263. 10.1007/s10584-016-1617-1 27471332 PMC4944792

[gh270063-bib-0040] Hosmer Jr, D. W. , Lemeshow, S. , & Sturdivant, R. X. (2013). Applied logistic regression. John Wiley and Sons.

[gh270063-bib-0041] Hutton, G. (2012). Global costs and benefits of reaching universal coverage of sanitation and drinking‐water supply. Journal of Water and Health, 11(1), 1–12. 10.2166/wh.2012.105 23428544

[gh270063-bib-0042] Islam, M. A. , Hoque, M. A. , Ahmed, K. M. , & Butler, A. P. (2019). Impact of climate change and land use on groundwater salinization in Southern Bangladesh—Implications for other Asian deltas. Environmental Management, 64(5), 640–649. 10.1007/s00267-019-01220-4 31655864

[gh270063-bib-0043] Jonell, T. N. , Giosan, L. , Clift, P. D. , Carter, A. , Bretschneider, L. , Hathorne, E. C. , et al. (2022). No modern Irrawaddy River until the late Miocene‐Pliocene. Earth and Planetary Science Letters, 584, 117516. 10.1016/j.epsl.2022.117516

[gh270063-bib-0044] Khan, A. E. , Ireson, A. , Kovats, S. , Mojumder, S. K. , Khusru, A. , Rahman, A. , & Vineis, P. (2011). Drinking water salinity and maternal health in coastal Bangladesh: Implications of climate change. Environmental Health Perspectives, 119(9), 1328–1332. 10.1289/ehp.1002804 21486720 PMC3230389

[gh270063-bib-0045] Klump, S. , Kipfer, R. , Cirpka, O. A. , Harvey, C. F. , Brennwald, M. S. , Ashfaque, K. N. , et al. (2006). Groundwater dynamics and arsenic mobilization in Bangladesh assessed using noble gases and tritium. Environmental Science and Technology, 40(1), 243–250. 10.1021/es051284w 16433358

[gh270063-bib-0046] Kmec, J. , & Šír, M. (2024). Modeling 2D gravity‐driven flow in unsaturated porous media for different infiltration rates. Hydrology and Earth System Sciences, 28(22), 4947–4970. 10.5194/hess-28-4947-2024

[gh270063-bib-0047] Kravtsova, V. I. , Mikhailov, V. N. , & Kidyaeva, V. M. (2009). Hydrological regime, morphological features and natural territorial complexes of the Irrawaddy River Delta (Myanmar). Water Resources, 36(3), 243–260. 10.1134/s0097807809030014

[gh270063-bib-0048] Kumar, S. , Ghosh, D. , Donselaar, M. E. , Burgers, F. , & Ghosh, A. K. (2021). Clay‐plug sediment as the locus of arsenic pollution in Holocene alluvial‐plain aquifers. Catena, 202, 105255. 10.1016/j.catena.2021.105255

[gh270063-bib-0049] Ledford, H. (2008). Irrawaddy may be poisoned by arsenic. Nature, 454(7202), 263‐263. 10.1038/454263a 18633380

[gh270063-bib-0050] Lovley, D. R. , & Anderson, R. T. (2000). Influence of dissimilatory metal reduction on fate of organic and metal contaminants in the subsurface. Hydrogeology Journal, 8(1), 77–88. 10.1007/PL00010974

[gh270063-bib-0051] Lowers, H. A. , Breit, G. N. , Foster, A. L. , Whitney, J. , Yount, J. , Uddin, M. N. , & Muneem, A. A. (2007). Arsenic incorporation into authigenic pyrite, Bengal Basin sediment, Bangladesh. Geochimica et Cosmochimica Acta, 71(11), 2699–2717. 10.1016/j.gca.2007.03.022

[gh270063-bib-0052] Mailloux, B. J. , Trembath‐Reichert, E. , Cheung, J. , Watson, M. , Stute, M. , Freyer, G. A. , et al. (2013). Advection of surface‐derived organic carbon fuels microbial reduction in Bangladesh groundwater. Proceedings of the National Academy of Sciences, 110(14), 5331–5335. 10.1073/pnas.1213141110 PMC3619377 23487743

[gh270063-bib-0053] McArthur, J. M. , Banerjee, D. M. , Hudson‐Edwards, K. A. , Mishra, R. , Purohit, R. , Ravenscroft, P. , et al. (2004). Natural organic matter in sedimentary basins and its relation to arsenic in anoxic groundwater: The example of West Bengal and its worldwide implications. Applied Geochemistry, 19(8), 1255–1293. 10.1016/j.apgeochem.2004.02.001

[gh270063-bib-0054] McArthur, J. M. , Nath, B. , Banerjee, D. M. , Purohit, R. , & Grassineau, N. (2011). Palaeosol control on groundwater flow and pollutant distribution: The example of arsenic. Environmental Science and Technology, 45(4), 1376–1383. 10.1021/es1032376 21268629

[gh270063-bib-0055] McArthur, J. M. , Ravenscroft, P. , Banerjee, D. M. , Milsom, J. , Hudson‐Edwards, K. A. , Sengupta, S. , et al. (2008). How paleosols influence groundwater flow and arsenic pollution: A model from the Bengal Basin and its worldwide implication. Water Resources Research, 44(11), W11411. 10.1029/2007WR006552

[gh270063-bib-0056] Meharg, A. A. , & Raab, A. (2010). Getting to the bottom of arsenic standards and guidelines. Environmental Science and Technology, 44(12), 4395–4399. 10.1021/es9034304 20465302

[gh270063-bib-0057] Meharg, A. A. , Scrimgeour, C. , Hossain, S. A. , Fuller, K. , Cruickshank, K. , Williams, P. N. , & Kinniburgh, D. G. (2006). Codeposition of organic carbon and arsenic in Bengal Delta aquifers. Environmental Science and Technology, 40(16), 4928–4935. 10.1021/es060722b 16955888

[gh270063-bib-0058] Michael, H. A. , & Voss, C. I. (2009). Controls on groundwater flow in the Bengal Basin of India and Bangladesh: Regional modeling analysis. Hydrogeology Journal, 17(7), 1561–1577. 10.1007/s10040-008-0429-4

[gh270063-bib-0059] Moore, O. C. , Holt, A. D. , Richards, L. A. , McKenna, A. M. , Spencer, R. G. M. , Lapworth, D. J. , et al. (2024). Characterisation of dissolved organic matter in two contrasting arsenic‐prone sites in Kandal Province, Cambodia. Organic Geochemistry, 198, 104886. 10.1016/j.orggeochem.2024.104886

[gh270063-bib-0060] Mukherjee, A. , Sarkar, S. , Coomar, P. , & Bhattacharya, P. (2023). Towards clean water: Managing risk of arsenic‐contaminated groundwater for human consumption. Current Opinion in Environmental Science & Health, 36, 100509. 10.1016/j.coesh.2023.100509

[gh270063-bib-0061] Mukherjee, A. , von Brömssen, M. , Scanlon, B. R. , Bhattacharya, P. , Fryar, A. E. , Hasan, M. A. , et al. (2008). Hydrogeochemical comparison and effects of overlapping redox zones on groundwater arsenic near the Western (Bhagirathi sub‐basin, India) and Eastern (Meghna sub‐basin, Bangladesh) margins of the Bengal Basin. Journal of Contaminant Hydrology, 99(1–4), 31–48. 10.1016/j.jconhyd.2007.10.005 18164513

[gh270063-bib-0062] Nath, B. , Das, A. , Majumder, S. , Roychowdhury, T. , Ni‐Meister, W. , & Rahman, M. M. (2022). Geospatial machine learning prediction of arsenic distribution in the groundwater of Murshidabad District, West Bengal, India: Analyzing spatiotemporal patterns to understand human health risk. ACS ES&T Water, 2(12), 2409–2421. 10.1021/acsestwater.2c00263

[gh270063-bib-0063] Neumann, R. B. , Ashfaque, K. N. , Badruzzaman, A. B. M. , Ashraf Ali, M. , Shoemaker, J. K. , & Harvey, C. F. (2010). Anthropogenic influences on groundwater arsenic concentrations in Bangladesh. Nature Geoscience, 3(1), 46–52. 10.1038/ngeo685

[gh270063-bib-0064] Nickson, R. , McArthur, J. M. , Burgess, W. G. , Ahmed, K. M. , Ravenscroft, P. , & Rahman, M. (1998). Arsenic poisoning in Bangladesh groundwater. Nature, 395(6700), 338. 10.1038/26387 9759723

[gh270063-bib-0065] Nwankwo, C. B. , Hoque, M. A. , Islam, M. A. , & Dewan, A. (2020). Groundwater constituents and trace elements in the basement aquifers of Africa and sedimentary aquifers of Asia: Medical hydrogeology of drinking water minerals and toxicants. Earth Systems and Environment, 4(2), 369–384. 10.1007/s41748-020-00151-z

[gh270063-bib-0066] Phyu, P. K. (2019). Arsenic contamination in underground water in Myanmar. In Paper presented at the Myanmar–UK project kick‐off symposium on rural water supply in the Ayeyarwady Delta. Hinthada University.

[gh270063-bib-0067] Rahman, A. , Mondal, N. C. , & Fauzia, F. (2021). Arsenic enrichment and its natural background in groundwater at the proximity of active floodplains of Ganga River, northern India. Chemosphere, 265, 129096. 10.1016/j.chemosphere.2020.129096 33280841

[gh270063-bib-0068] Ravenscroft, P. , Brammer, H. , & Richards, K. S. (2009). Arsenic pollution: A global synthesis (1st ed.). Wiley‐Blackwell.

[gh270063-bib-0069] Ravenscroft, P. , McArthur, J. M. , & Hoque, M. A. (2013). Stable groundwater quality in deep aquifers of Southern Bangladesh: The case against sustainable abstraction. Science of the Total Environment, 454–455, 627–638. 10.1016/j.scitotenv.2013.02.071 23584139

[gh270063-bib-0070] Scheelbeek, P. F. D. , Chowdhury, M. A. H. , Haines, A. , Alam, D. S. , Hoque, M. A. , Butler, A. P. , et al. (2017). Drinking water salinity and raised blood pressure: Evidence from a cohort Study in coastal Bangladesh. Environmental Health Perspectives, 125(5), 057007‐057001. 10.1289/EHP659 28599268 PMC5730519

[gh270063-bib-0071] Shamsudduha, M. , Marzen, L. J. , Uddin, A. , Lee, M.‐K. , & Saunders, J. A. (2009). Spatial relationship of groundwater arsenic distribution with regional topography and water‐table fluctuations in the shallow aquifers in Bangladesh. Environmental Geology, 57(7), 1521–1535. 10.1007/s00254-008-1429-3

[gh270063-bib-0072] Sharma, A. (2017). Drinking water quality in Indian water policies, laws, and courtrooms: Understanding the intersections of science and law in developing countries. Bulletin of Science, Technology and Society, 37(1), 45–56. 10.1177/0270467617738696

[gh270063-bib-0073] Sieczko, A. , & Peduzzi, P. (2014). Origin, enzymatic response and fate of dissolved organic matter during flood and non‐flood conditions in a river‐floodplain system of the Danube (Austria). Aquatic Sciences, 76(1), 115–129. 10.1007/s00027-013-0318-3 24415892 PMC3883529

[gh270063-bib-0074] Stahl, M. O. , Harvey, C. F. , van Geen, A. , Sun, J. , Thi Kim Trang, P. , Mai Lan, V. , et al. (2016). River bank geomorphology controls groundwater arsenic concentrations in aquifers adjacent to the Red River, Hanoi Vietnam. Water Resources Research, 52(8), 6321–6334. 10.1002/2016WR018891

[gh270063-bib-0075] Stute, M. , Zheng, Y. , Schlosser, P. , Horneman, A. , Dhar, R. K. , Hoque, M. A. , et al. (2007). Hydrological control of as concentrations in Bangladesh groundwater. Water Resources Research, 43(9), W09417. 10.1029/2005WR004499

[gh270063-bib-0076] Tóth, J. (1963). A theoretical analysis of groundwater flow in small drainage basins. Journal of Geophysical Research, 68(16), 4795–4812. 10.1029/jz068i016p04795

[gh270063-bib-0077] Tripartite Core Group . (2008). Post‐Nargis Periodic review I. United Nations (UN) in Myanmar. Retrieved from https://www.gfdrr.org/sites/default/files/GFDRR_Myanmar_Post‐Nargis_Joint_Assessment_2008_EN.pdf

[gh270063-bib-0078] Tsai, C. , Hoque, M. A. , Vineis, P. , Ahmed, K. M. , & Butler, A. P. (2024). Salinisation of drinking water ponds and groundwater in coastal Bangladesh linked to tropical cyclones. Scientific Reports, 14(1), 5211. 10.1038/s41598-024-54446-6 38433257 PMC10909877

[gh270063-bib-0079] Tun, T. N. (2003). Arsenic contamination of water sources in rural Myanmar. In P. Harvey (ed.), Towards the millennium development goals ‐ Actions for water and environmental sanitation: Proceedings of the 29th WEDC International Conference (pp. 219–221).

[gh270063-bib-0080] van Geen, A. , Aziz, Z. , Horneman, A. , Weinman, B. , Dhar, R. K. , Zheng, Y. , et al. (2006). Preliminary evidence of a link between surface soil properties and the arsenic content of shallow groundwater in Bangladesh. Journal of Geochemical Exploration, 88(1–3), 157–161. 10.1016/j.gexplo.2005.08.106

[gh270063-bib-0081] van Geen, A. , Win, K. H. , Zaw, T. , Naing, W. , Mey, J. L. , & Mailloux, B. (2014). Confirmation of elevated arsenic levels in groundwater of Myanmar. Science of the Total Environment, 478, 21–24. 10.1016/j.scitotenv.2014.01.073 24530581 PMC3954970

[gh270063-bib-0082] van Geen, A. , Zhang, Y. , Goodbred, S. , Horneman, A. , Aziz, Z. , Cheng, Z. , et al. (2008). Flushing history as a hydrogeological control on the regional distribution of arsenic in shallow groundwater of the Bengal basin. Environmental Science and Technology, 42(7), 2283–2288. 10.1021/es702316k 18504954 PMC3050603

[gh270063-bib-0083] Vogel, A. , Seeger, K. , Brill, D. , Brückner, H. , Kyaw, A. , Myint, Z. N. , & Kraas, F. (2024). Towards integrated flood management: Vulnerability and flood risk in the Ayeyarwady Delta of Myanmar. International Journal of Disaster Risk Reduction, 114, 104723. 10.1016/j.ijdrr.2024.104723

[gh270063-bib-0084] Wallis, I. , Prommer, H. , Berg, M. , Siade, A. J. , Sun, J. , & Kipfer, R. (2020). The river–groundwater interface as a hotspot for arsenic release. Nature Geoscience, 13(4), 288–295. 10.1038/s41561-020-0557-6

[gh270063-bib-0085] Ward, R. C. (1963). The effect of site factors on water‐table fluctuations. Journal of Hydrology, 1(2), 151–165. 10.1016/0022-1694(63)90038-6

[gh270063-bib-0086] Weber, K. A. , Achenbach, L. A. , & Coates, J. D. (2006). Microorganisms pumping iron: Anaerobic microbial iron oxidation and reduction. Nature Reviews Microbiology, 4(10), 752–764. 10.1038/nrmicro1490 16980937

[gh270063-bib-0087] Wen, H. , Perdrial, J. , Abbott, B. W. , Bernal, S. , Dupas, R. , Godsey, S. E. , et al. (2020). Temperature controls production but hydrology regulates export of dissolved organic carbon at the catchment scale. Hydrology and Earth System Sciences, 24(2), 945–966. 10.5194/hess-24-945-2020

[gh270063-bib-0088] Winkel, L. , Berg, M. , Amini, M. , Hug, S. J. , & Annette Johnson, C. (2008). Predicting groundwater arsenic contamination in Southeast Asia from surface parameters. Nature Geoscience, 1(8), 536–542. 10.1038/ngeo254

[gh270063-bib-0089] Yuan, X. , Deng, Y. , Du, Y. , Xue, J. , Pi, K. , Yang, Y. , et al. (2025). Processing pathways of organic matter under methanogenic conditions and its influence on arsenic mobilization in aquifers. Journal of Hydrology, 647, 132367. 10.1016/j.jhydrol.2024.132367

[gh270063-bib-0090] Zijl, W. (1999). Scale aspects of groundwater flow and transport systems. Hydrogeology Journal, 7(1), 139–150. 10.1007/s100400050185

