# Supplementary material for: Mapping Arsenic Risks in the Ayeyarwady (Irrawaddy) Delta, Myanmar: Implications for Public Health
Source: Geohealth. 2025 Oct 29;9(11):e2024GH001326. doi: 10.1029/2024GH001326 (PMC12569530; doi:10.1029/2024GH001326)
Supplement: Supplementary file 1 — Supporting Information S1 [file GH2-9-e2024GH001326-s001.docx]

*GeoHealth*

Supporting Information for

**Mapping Arsenic Risks in the Ayeyarwady (Irrawaddy) Delta, Myanmar: Implications for Public Health**

*M. A. Hoque^1,*^, K. K. Khaing^2^, M. Fowler^1^, M. S. Sultana^1^, C. C. Myint^2^, A. Swe^3^, P. Dennis^4^, S. Shahid^5^, G. R. Fones^1^*

^1^School of the Environment and Life Sciences (SELS), University of Portsmouth, Portsmouth, UK

^2^Department of Geography, Hinthada University, Ayeyarwady Region, Myanmar

^3^Department of Geography, University of Yangon, Myanmar

^4^School of Environmental Sciences, University of East Anglia, Norwich Research Park, Norwich, UK

^5^Department of Water & Environmental Engineering, Faculty of Civil Engineering, Universiti Teknologi Malaysia (UTM), Johor Bahru, Malaysia

* [mo.hoque@port.ac.uk](mailto:mo.hoque@port.ac.uk)

**Contents of this file**

**Fig. S1:** Groundwater recharge in this region is influenced by topographical variations and fluctuating water levels.

**Table S1:** Laboratory Analyses and Field Observations for 81 Samples.

**
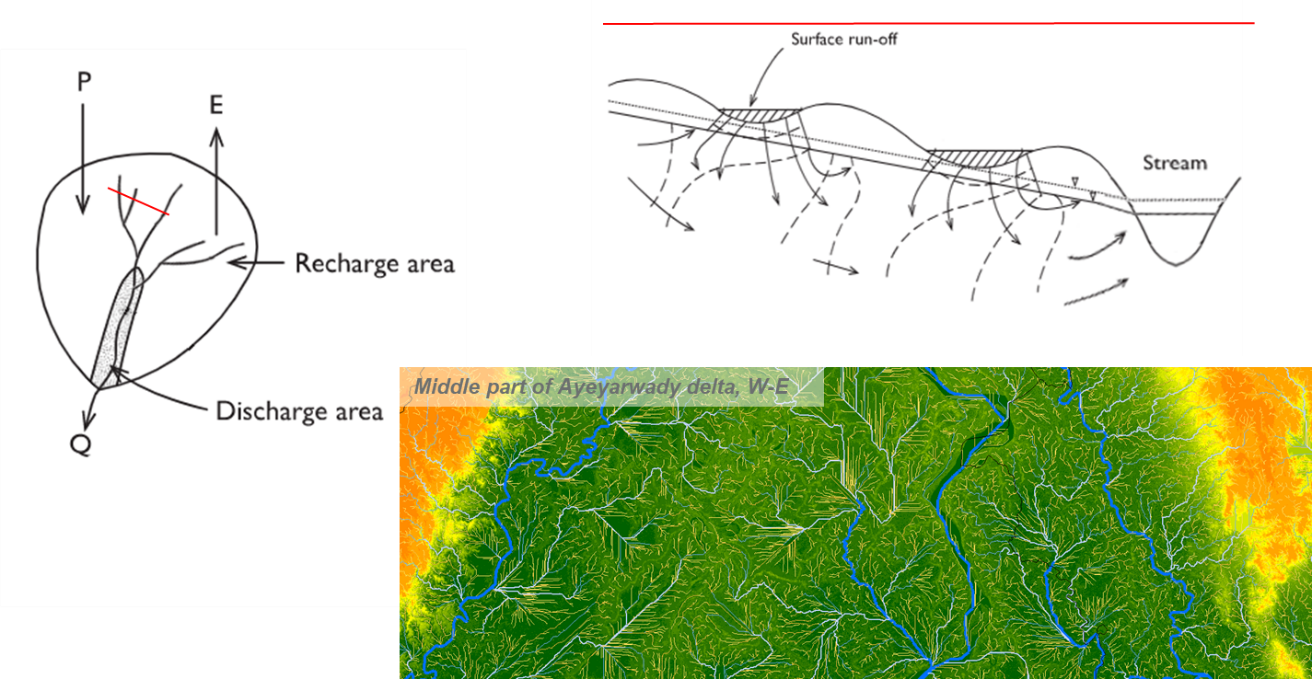
**

**Fig. S1:** Groundwater recharge in this region is influenced by topographical variations and fluctuating water levels. Recharge occurs predominantly in elevated microdepressions within individual catchments and is bidirectional, depending on the hydraulic head. During floods, low-lying areas experience halted recharge, with water influx concentrated in higher elevations via low-order streams and depressions. Post-flood, or early in the rainy season, these lower areas begin to recharge through rainfall and lower order streams. Conversely, larger rivers act as discharge zones in the regional topography, contributing to groundwater outflow under natural conditions. Recharge from these rivers is temporally limited and spatially confined, due to the downward gradient and rapid equilibrium between the river water and surrounding groundwater.

Top left: A typical hydrological catchment, recharge primarily occurs in micro-depressions at higher elevations.

Top right: A schematic representation of surface runoff and subsurface flow within a catchment, highlighting that during flooding, recharge is limited to higher elevation catchments and restricted largely to low-order streams and depressions.

Bottom: Satellite imagery of the middle part of the Ayeyarwady Delta from west to east, showing the intricate network of hierarchical catchments. Larger rivers act as regional discharge zones under natural conditions, with any recharge from them being temporally brief and spatially limited due to the downward gradient and equilibrium with surrounding groundwater.

**Table S1:** Laboratory Analyses and Field Observations for 81 Samples. This table presents detailed laboratory results for 81 groundwater samples collected from the Ayeyarwady Delta. It includes the concentration of arsenic and other relevant chemical parameters measured. Each entry is accompanied by GPS coordinates, depth information, and specific field observations to provide a complete contextual understanding of each sample's environmental setting.

Part 1:

| SL | Sample ID | Depth_ft | Depth_m | Installation | d18Ovsmow | dD vsmow | Na_ppm | K_ppm | Ca_ppm | Mg_ppm | Fe_ppb | Mn_ppb | Si_ppb | Sr_ppb | Ba_ppb | As_ppb_4 |
| --- | --- | --- | --- | --- | --- | --- | --- | --- | --- | --- | --- | --- | --- | --- | --- | --- |
| 1 | W1/01 | 60 | 18 | 2016 | -4.3 | -25.09 | 24.226 | 5.417 | 9.784 | 19.859 | 3312.17 | 768.039 | 57950.7 | 114.477 | 79.306 | 4 |
| 2 | W1/02 | 170 | 52 | 2018 | -4.91 | -27.22 | 5.341 | 4.009 | 16.195 | 3.197 | 39.162 | 6.133 | 64036.2 | 100.524 | 5.285 | 4 |
| 3 | W1/03 | 160 | 49 | 1985 | -5.47 | -32.13 | 106.686 | 7.999 | 61.405 | 33.646 | 2535.58 | 279.2 | 67459.1 | 564.662 | 76.421 | 4 |
| 4 | W1/04 | 180 | 55 | 2019 | -5.33 | -32.18 | 210.408 | 25.132 | 26.385 | 50.999 | 187.652 | 2578.22 | 25147.2 | 358.584 | 388.104 | 4 |
| 5 | W1/05 | 80 | 24 | 2013 | -6.98 | -45.53 | 24.452 | 2.561 | 5.209 | 5.33 | 20.151 | 449.525 | 65288.3 | 43.56 | 2 | 5.412 |
| 6 | W10/01 | 30 | 9 | 2009 | -5.5 | -30.56 | 0.004 | 2.833 | 40.494 | 17.844 | 241.676 | 2339 | 39999.3 | 233.592 | 36.038 | 4 |
| 7 | W10/02 | 45 | 14 | 2017 | -5.6 | -36.1 | 0.004 | 1.77 | 31.81 | 19.734 | 912.076 | 5152.58 | 41863 | 210.377 | 32.408 | 4 |
| 8 | W10/03 | 80 | 24 | 2011 | -4.52 | -23.09 | 0.004 | 2.169 | 39.671 | 29.678 | 2746.22 | 2886.19 | 36251.2 | 249.613 | 52.646 | 4 |
| 9 | W10/04 | 60 | 18 | 2009 | -4.6 | -23.84 | 0.004 | 3.808 | 82.72 | 36.644 | 2312.71 | 2052.52 | 42889.9 | 537.346 | 79.439 | 26.863 |
| 10 | W10/05 | 140 | 43 | 2017 | -4.47 | -22.94 | 96.947 | 5.449 | 27.441 | 19.977 | 306.93 | 120.644 | 26572.9 | 302.732 | 149.597 | 28.72 |
| 11 | W11/01 | 15 | 5 | 2017 | -4.68 | -25.03 | 146.322 | 5.849 | 29.316 | 23.7 | 4523.15 | 321.778 | 23259.7 | 327.158 | 177.21 | 4 |
| 12 | W11/02 | 150 | 46 | 2018 | -4.71 | -25.53 | 149.682 | 5.241 | 46.796 | 37.824 | 2315.44 | 670.119 | 38617.4 | 488.859 | 221.538 | 4 |
| 13 | W11/03 | 60 | 18 | 2014 | -5.1 | -27.66 | 344.983 | 20.495 | 24.916 | 56.799 | 51.8 | 63.093 | 40696.5 | 453.248 | 36.609 | 87.751 |
| 14 | W11/04 | 160 | 49 | 2008 | -4.78 | -25.52 | 155.437 | 5.757 | 35.525 | 26.091 | 1106.73 | 265.719 | 33720.7 | 373.254 | 166.824 | 4 |
| 15 | W11/05 | 180 | 55 | 2009 | -4.67 | -24.86 | 140.277 | 4.989 | 40.944 | 33.409 | 7.794 | 677.811 | 38032.4 | 449.113 | 219.447 | 4 |
| 16 | W12/01 | 25 | 8 | 1994 | -6.36 | -37.88 | 14.515 | 2.759 | 39.282 | 29.474 | 143.852 | 2112.93 | 40984.7 | 204.195 | 52.278 | 4 |
| 17 | W12/02 | 300 | 91 | 2005 | -4.62 | -25.5 | 72.219 | 8.506 | 14.249 | 18.477 | 405.607 | 508.15 | 56432.1 | 186.652 | 66.755 | 4 |
| 18 | W12/03 | 35 | 11 | 2009 | -6.47 | -38.91 | 104.233 | 8.736 | 60.221 | 37.019 | 70.587 | 181.49 | 32940.5 | 448.868 | 113.622 | 76.483 |
| 19 | W12/04 | 80 | 24 | 2004 | -5.27 | -30.05 | 33.361 | 4.296 | 132.702 | 55.088 | 23.631 | 1908.45 | 35405.5 | 774.653 | 152.279 | 108.123 |
| 20 | W12/05 | 135 | 41 | 2016 | -5.77 | -34.15 | 211.39 | 20.901 | 24.024 | 31.64 | 27.851 | 182.526 | 21185.4 | 276.02 | 300.827 | 4 |
| 21 | W13/01 | 125 | 38 | 2016 | -7.08 | -43.49 | 57.804 | 4.052 | 0.002 | 0.538 | 75.604 | 4 | 25028 | 5.731 | 8.245 | 18.764 |
| 22 | W13/02 | 130 | 40 | 2018 | -7.68 | -46.92 | 62.892 | 4.234 | 0.002 | 0.582 | 60.51 | 5.406 | 25599.5 | 6.188 | 9.579 | 30.317 |
| 23 | W13/03 | 130 | 40 | 2016 | -6.65 | -41.96 | 330.645 | 22.434 | 3.664 | 13.833 | 56.968 | 11.788 | 26856.2 | 104.953 | 113.315 | 29.992 |
| 24 | W13/04 | 130 | 40 | 2014 | -6.84 | -42.31 | 324.531 | 21.836 | 3.423 | 12.797 | 29.437 | 43.963 | 28503.8 | 99.503 | 87.959 | 17.725 |
| 25 | W14/01 | 100 | 30 | 2016 | -4.63 | -26.76 | 97.129 | 28.017 | 1.851 | 6.795 | 29.143 | 58.992 | 30949.5 | 63.311 | 18.902 | 4 |
| 26 | W14/02 | 100 | 30 | 2006 | -4.61 | -26.21 | 151.546 | 14.586 | 0.767 | 4.204 | 59.161 | 36.402 | 15111.9 | 36.404 | 83.904 | 6.218 |
| 27 | W14/03 | 120 | 37 | 2014 | -4.82 | -26.92 | 150.672 | 14.285 | 1.842 | 5.958 | 395.268 | 208.352 | 15956.7 | 40.514 | 75.575 | 8.066 |
| 28 | W14/04 | 120 | 37 | 2014 | -4.6 | -25.87 | 167.467 | 15.067 | 1.078 | 5.259 | 12.05 | 80.41 | 16074.2 | 44.035 | 52.375 | 4 |
| 29 | W14/05 | 110 | 34 | 2015 | -4.7 | -26.25 | 125.891 | 10.555 | 0.728 | 3.271 | 9.038 | 37.15 | 19766.6 | 28.424 | 48.111 | 4 |
| 30 | W15/01 | 140 | 43 | 2015 | -4.96 | -30.17 | 246.223 | 15.221 | 17.46 | 27.776 | 26.802 | 547.651 | 33078.9 | 183.995 | 23.066 | 4 |
| 31 | W15/02 | 120 | 37 | 2010 | -4.75 | -27.47 | 172.211 | 10.294 | 8.919 | 16.551 | 12.524 | 269.187 | 34346.4 | 97.622 | 28.253 | 7.509 |
| 32 | W15/03 | 140 | 43 | 2009 | -4.69 | -29.35 | 335.18 | 24.714 | 46.674 | 62.444 | 170.059 | 1745.43 | 36016.9 | 522.58 | 45.012 | 4 |
| 33 | W15/04 | 180 | 55 | 2004 | -4.97 | -29.89 | 364.046 | 44.609 | 69.296 | 92.94 | 14.963 | 3260.24 | 23095.5 | 935.32 | 404.924 | 4 |
| 34 | W15/05 | 160 | 49 | 2011 | -5.25 | -31.9 | 492.07 | 28.172 | 108.057 | 94.54 | 94.602 | 4532.65 | 44440.7 | 1028.28 | 69.012 | 4.088 |
| 35 | W16/01 | 360 | 110 | 2015 | -4.02 | -22.97 | 3.891 | 0.973 | 2.626 | 0.148 | 48.161 | 71.109 | 8215.1 | 10.118 | 21.769 | 4 |
| 36 | W16/02 | 350 | 107 | 2016 | -4.19 | -24.33 | 4.434 | 1.316 | 0.002 | 0.002 | 8.783 | 118.203 | 10375.3 | 5.274 | 38.949 | 4 |
| 37 | W16/03 | 300 | 91 | 2019 | -3.79 | -22.27 | 3.732 | 1.004 | 4.202 | 0.002 | 10.336 | 108.52 | 11355.3 | 5.632 | 21.364 | 4.605 |
| 38 | W16/04 | 260 | 79 | 2015 | -3.89 | -22.35 | 1.257 | 7.52 | 3.502 | 1.024 | 9.903 | 4 | 30245.5 | 29.103 | 75.689 | 5.134 |
| 39 | W2/01 | 170 | 52 | 2014 | -3.8 | -17.11 | 0.004 | 2.886 | 1.752 | 3.505 | 7.255 | 29.727 | 15727.7 | 25.593 | 44.216 | 4 |
| 40 | W2/02 | 260 | 79 | 2017 | -4.92 | -27.84 | 20.959 | 4.522 | 10.148 | 7.233 | 21.675 | 199.415 | 60237.2 | 121.297 | 21.485 | 4 |
| 41 | W2/03 | 5 | 2 | 2004 | -4.52 | -24.35 | 0.004 | 7.504 | 0.307 | 0.154 | 47.532 | 53.947 | 10030.7 | 0.758 | 32.535 | 4 |
| 42 | W2/04 | 120 | 37 | 2016 | -5.16 | -29.2 | 14.331 | 4.924 | 0.772 | 1.327 | 7.854 | 103.745 | 18775.9 | 11.862 | 88.095 | 4 |
| 43 | W2/05 | 200 | 61 | 2019 | -4.99 | -28.04 | 23.272 | 5.527 | 12.34 | 10.64 | 65.538 | 442.906 | 38727.5 | 157.846 | 117.663 | 4 |
| 44 | W2/06 | 95 | 29 | 2016 | -4.33 | -25.47 | 11.658 | 4.814 | 3.309 | 2.958 | 8.785 | 36.942 | 16342.4 | 42.304 | 128.866 | 4 |
| 45 | W3/01 | 40 | 12 | 1915 | -4.96 | -28.6 | 6.616 | 0.404 | 7.079 | 8.581 | 3319.7 | 152.695 | 30783 | 41.956 | 41.429 | 9.438 |
| 46 | W3/02 | 40 | 12 | 2013 | -4.59 | -23.89 | 3.319 | 2.611 | 6.766 | 6.566 | 402.104 | 125.257 | 32086.3 | 52.558 | 15.109 | 4 |
| 47 | W3/03 | 60 | 18 | 2016 | -3.68 | -19.06 | 61.29 | 1.092 | 23.508 | 34.742 | 30902.7 | 1908.04 | 29975.6 | 134.449 | 147.195 | 5.142 |
| 48 | W3/04 | 37 | 11 | 2018 | -4.53 | -26.1 | 5.203 | 0.588 | 6.159 | 7.593 | 8583.79 | 194.527 | 33673.1 | 42.811 | 37.49 | 7.006 |
| 49 | W3/05 | 360 | 110 | 2016 | -5.11 | -28.48 | 7.707 | 6.951 | 9.329 | 13.771 | 32.886 | 890.064 | 37837.9 | 130.809 | 323.492 | 4 |
| 50 | W3/06 | 180 | 55 | 2004 | -5.72 | -34.1 | 26.023 | 1.654 | 17.145 | 13.988 | 34799.3 | 1475.3 | 48091.7 | 158.765 | 98.282 | 4.797 |
| 51 | W4/01 | 250 | 76 | 2014 | -5.92 | -35.13 | 68.586 | 2.265 | 44.021 | 52.959 | 18.959 | 1265.11 | 35032 | 342.127 | 47.213 | 4 |
| 52 | W4/02 | 220 | 67 | 2019 | -6.8 | -42.87 | 76.509 | 2.543 | 51.179 | 57.692 | 20.399 | 1472.02 | 35389.2 | 397.144 | 49.452 | 4 |
| 53 | W4/03 | 180 | 55 | 1999 | -3.45 | -15.85 | 67.445 | 2.853 | 72.709 | 82.482 | 148.581 | 102.724 | 39697.6 | 672.657 | 150.094 | 134.995 |
| 54 | W4/04 | 600 | 183 | 2014 | -5.28 | -30.44 | 27.346 | 2.963 | 10.933 | 6.644 | 1500.54 | 331 | 63966.3 | 116.984 | 58.62 | 4 |
| 55 | W4/05 | 240 | 73 | 2013 | -5.24 | -28.9 | 63.301 | 2.813 | 39.693 | 45.882 | 5.475 | 404.04 | 34283.1 | 349.982 | 115.346 | 4 |
| 56 | W5/01 | 220 | 67 | 2019 | -6.17 | -37.99 | 39.863 | 3.672 | 45.461 | 36.615 | 5607.8 | 190.612 | 36740.4 | 477.43 | 140.543 | 97.346 |
| 57 | W5/02 | 195 | 59 | 2018 | -6.31 | -38.66 | 40.602 | 4.402 | 48.794 | 42.082 | 2843.97 | 1726.71 | 35636.6 | 531.748 | 198.514 | 416.125 |
| 58 | W5/03 | 700 | 213 | 2018 | -5.89 | -37.07 | 220.698 | 8.882 | 82.338 | 65.046 | 47.159 | 3095.68 | 55053 | 850.74 | 368.145 | 4 |
| 59 | W5/04 | 180 | 55 | 1996 | -6.08 | -37.56 | 36.872 | 3.561 | 45.142 | 33.155 | 5039.27 | 368.129 | 42760.3 | 364.085 | 96.887 | 48.187 |
| 60 | W5/05 | 180 | 55 | 2016 | -6.13 | -37.94 | 39.635 | 3.797 | 56.817 | 40.809 | 72.075 | 1331.39 | 37989.3 | 583.955 | 92 | 4 |
| 61 | W6/01 | 180 | 55 | 2011 | -4.07 | -24.02 | 26.055 | 2.203 | 22.251 | 12.894 | 64.726 | 263.156 | 44857.8 | 181.685 | 79.266 | 4 |
| 62 | W6/02 | 180 | 55 | 2013 | -4.62 | -26.14 | 11.185 | 1.725 | 22.22 | 12.647 | 4740.57 | 362.915 | 52232.6 | 163.416 | 21.382 | 60.219 |
| 63 | W6/03 | 300 | 91 | 2016 | -7.08 | -44.58 | 18.885 | 2.635 | 41.883 | 25.178 | 23.168 | 1292.46 | 46676.9 | 396.678 | 87.641 | 12.116 |
| 64 | W6/04 | 380 | 116 | 2015 | -6.7 | -41.74 | 41.224 | 2.368 | 19.274 | 12.362 | 206.758 | 774.586 | 57357.4 | 193.318 | 90.096 | 4 |
| 65 | W6/05 | 400 | 122 | 2016 | -4.08 | -23.87 | 25.525 | 2.304 | 22.525 | 13.126 | 2.235 | 4 | 45027.3 | 183.972 | 73.069 | 4 |
| 66 | W6/06 | 20 | 6 | 1996 | -5.63 | -32.19 | 40.968 | 15.406 | 47.648 | 36.049 | 165.811 | 202.583 | 23357.2 | 256.426 | 106.262 | 4 |
| 67 | W7/01 | 105 | 32 | 2018 | -4.65 | -25.38 | 5.833 | 0.479 | 14.237 | 10.603 | 12504.9 | 651.532 | 54945.3 | 101.006 | 82.987 | 44.132 |
| 68 | W7/02 | 125 | 38 | 1990 | -4.08 | -23.05 | 7.428 | 1.123 | 24.048 | 14.089 | 10048.3 | 629.241 | 57224 | 168.419 | 44.764 | 47.169 |
| 69 | W7/03 | 140 | 43 | 2000 | -4.81 | -26.65 | 8.287 | 3.071 | 14.905 | 23.737 | 4435.64 | 351.373 | 52374.7 | 138.076 | 44.867 | 66.535 |
| 70 | W7/04 | 330 | 101 | 2018 | -4.78 | -26.39 | 22.361 | 1.904 | 16.4 | 12.896 | 213.2 | 518.587 | 47837.5 | 99.788 | 14.764 | 4 |
| 71 | W7/05 | 30 | 9 | 2012 | -4.04 | -21.64 | 6.011 | 0.08 | 6.34 | 5.673 | 105.515 | 6.641 | 33221.5 | 43.893 | 5.084 | 4 |
| 72 | W8/1 | 140 | 43 | 2015 | -5.16 | -29.11 | 20.56 | 1.176 | 19.119 | 13.243 | 49.057 | 696.658 | 33550.6 | 153.425 | 25.621 | 4 |
| 73 | W8/2 | 210 | 64 | 2016 | -5.1 | -28.83 | 15.333 | 1.337 | 25.079 | 14.054 | 22.33 | 1447.95 | 37128.9 | 223.572 | 30.838 | 4 |
| 74 | W8/3 | 150 | 46 | 2015 | -5.08 | -29.04 | 23.024 | 1.208 | 21.71 | 15.256 | 138.161 | 724.803 | 33321 | 171.681 | 26.162 | 4 |
| 75 | W8/4 | 180 | 55 | 2004 | -5.11 | -29.76 | 23.114 | 1.421 | 29.224 | 19.045 | 30.232 | 811.333 | 37078.3 | 223.193 | 43.271 | 4 |
| 76 | W8/5 | 155 | 47 | 2004 | -5.14 | -28.94 | 30.206 | 1.753 | 23.27 | 17.902 | 85.472 | 540.249 | 38394.4 | 229.513 | 81.853 | 31.864 |
| 77 | W9/01 | 180 | 55 | 2019 | -5.28 | -29.33 | 97.567 | 2.295 | 33.752 | 18.509 | 60.008 | 1820.61 | 31444 | 296.423 | 49.943 | 4 |
| 78 | W9/02 | 190 | 58 | 2016 | -4.43 | -23.1 | 104.148 | 2.635 | 38.78 | 19.738 | 79.103 | 1400.37 | 32801 | 349.091 | 101.053 | 4 |
| 79 | W9/03 | 110 | 34 | 2019 | -4.43 | -23.08 | 100.274 | 2.398 | 37.437 | 19.465 | 48.751 | 1355.6 | 30996.8 | 340.836 | 108.643 | 4 |
| 80 | W9/04 | 180 | 55 | 2006 | -5.2 | -29.56 | 100.699 | 2.159 | 31.966 | 16.809 | 40.924 | 1495.99 | 26114 | 281.805 | 97.114 | 4 |
| 81 | W9/05 | 165 | 50 | 2007 | -4.98 | -27.06 | 96.906 | 1.784 | 34.152 | 17.602 | 952.231 | 1913.45 | 29502.9 | 299.644 | 188.199 | 10.982 |

Part 2:

| SL | Sample ID | Lat | Lon | Flood_Yes | Flood_No | Platform | Platform Colour | Utensil | ECuScm | pH | Eh | Temp | DO | TDS | HCO3 |
| --- | --- | --- | --- | --- | --- | --- | --- | --- | --- | --- | --- | --- | --- | --- | --- |
| 1 | W1/01 | 16.7972 | 94.7605 |  | N | Motor | Red | Red | 390 | 6.08 | 55 | 26.7 | 7 | 190 | 500 |
| 2 | W1/02 | 16.78763 | 94.74896 |  | N | Motor | Red | Red | 140 | 6.25 | 77 | 27.4 | 3.5 | 60 | 500 |
| 3 | W1/03 | 16.78665 | 94.73222 | y |  | Motor | Red | Black and Red | 1500 | 6.31 | 29 | 27.2 |  | 740 |  |
| 4 | W1/04 | 16.77686 | 94.73427 | y |  | Motor | no colour | no colour | 2460 | 4.94 | 140 | 26.8 | 2 | 1230 | 0 |
| 5 | W1/05 | 16.7737 | 94.72763 | y |  | Motor | Red | Black and Red | 250 | 6.62 | 102 | 27.1 |  | 120 |  |
| 6 | W10/01 | 17.01412 | 95.56505 | Y |  | Motor |  |  | 300 | 6.89 | 29 | 29.1 | 4 | 140 | 90 |
| 7 | W10/02 | 17.00659 | 95.56677 |  | N | Motor |  |  | 300 | 6.61 | 8 | 29.3 |  | 140 |  |
| 8 | W10/03 | 17.00561 | 95.56611 |  | N | Hand Pump | |  | 400 | 6.69 | -28 | 28.4 | 5 | 190 | 100 |
| 9 | W10/04 | 17.00571 | 95.5657 | Y |  | Hand Pump | |  | 590 | 7.23 | -93 | 28.4 | 4 | 290 | 70 |
| 10 | W10/05 | 17.04436 | 95.55119 | Y |  | Hand Pump | |  | 1230 | 7.14 | -20 | 28.6 | 5 | 610 | 100 |
| 11 | W11/01 | 16.97799 | 95.63464 |  | N | Motor | red | red | 1510 | 6.85 | -65 | 28.8 | 8 | 750 | 150 |
| 12 | W11/02 | 16.97842 | 95.63426 |  | N | Motor |  |  | 1650 | 6.96 | -41 | 28.9 | 5 | 820 | 90 |
| 13 | W11/03 | 16.9783 | 95.63434 |  | N | Motor | red | black and red | 3740 | 7.34 | 27 | 28.8 | 3 | 1870 | 60 |
| 14 | W11/04 | 16.97805 | 95.63463 |  | N | Motor | red | red | 1600 | 6.73 | -12 | 28.9 | 8 | 800 | 100 |
| 15 | W11/05 | 16.97799 | 95.63333 |  | N | Motor | red | red | 1600 | 7.23 | 48 | 28.8 |  | 800 |  |
| 16 | W12/01 | 16.98186 | 95.7308 |  | N | Motor |  |  | 430 | 6.83 | 48 | 29.1 | 5 | 210 | 75 |
| 17 | W12/02 | 16.97988 | 95.73024 |  | N | Motor |  |  | 800 | 6.88 | -19 | 29.1 | 6 | 390 | 500 |
| 18 | W12/03 | 16.98048 | 95.72961 |  | N | Hand Pump | |  | 1270 | 7.24 | 8 | 28.9 |  | 630 |  |
| 19 | W12/04 | 16.97996 | 95.72917 |  | N | Hand Pump | |  | 1040 | 7.17 | 60 | 28.7 | 4 | 520 | 60 |
| 20 | W12/05 | 16.97996 | 95.72894 |  | N | Motor |  |  | 1790 | 7.09 | 66 | 29.2 | 4 | 890 | 60 |
| 21 | W13/01 | 16.94094 | 95.84059 |  | N | Mortor |  |  | 460 | 8.21 | 81 | 27.4 | 3 | 220 | 200 |
| 22 | W13/02 | 16.94044 | 95.84989 |  | N | Mortor |  |  | 480 | 8.26 | 94 | 27.3 |  | 240 |  |
| 23 | W13/03 | 16.93852 | 95.84009 | Y |  | Mortor |  |  | 2890 | 7.75 | 114 | 27.3 | 5 | 1440 | 70 |
| 24 | W13/04 | 16.93822 | 95.84049 |  | N | Motor |  |  | 2840 | 7.66 | 117 | 27.7 | 2 | 1420 | 65 |
| 25 | W14/01 | 16.91339 | 95.94048 | Y |  | Motor |  |  | 1020 | 7.35 | 71 | 26.8 | 2 | 510 | 100 |
| 26 | W14/02 | 16.91511 | 95.99961 |  | N | Motor |  |  | 1350 | 7.45 | 113 | 27.5 |  | 670 |  |
| 27 | W14/03 | 16.91517 | 95.93386 |  | N | Motor |  |  | 1340 | 7.33 | 75 | 27.2 | 6 | 660 | 75 |
| 28 | W14/04 | 16.91434 | 95.93339 | Y | N | Motor |  |  | 1470 | 7.41 | 87 | 27.2 |  | 730 |  |
| 29 | W14/05 | 16.91307 | 95.93109 |  | N | Motor |  |  | 1070 | 7.53 | 106 | 27.5 | 4 | 530 | 80 |
| 30 | W15/01 | 16.86683 | 96.04493 |  | N | Motor |  |  | 2310 | 7.47 | 124 | 27.9 | 10 | 1150 | 80 |
| 31 | W15/02 | 16.86904 | 96.04478 |  | N | Hand Pump | |  | 1570 | 7.72 | 119 | 27.9 |  | 780 |  |
| 32 | W15/03 | 16.86969 | 96.04535 |  | N | Hand Pump | |  | 3490 | 6.67 | 65 | 28 | 3 | 1740 | 75 |
| 33 | W15/04 | 16.86591 | 96.05265 |  | N | Hand Pump | |  | 4250 | 6.99 | 77 | 28 | 8 | 2120 | 100 |
| 34 | W15/05 | 16.86895 | 96.04374 |  | N | Motor |  |  | 5690 | 6.22 | 100 | 27.6 | 4 | 2840 | 250 |
| 35 | W16/01 | 16.86159 | 96.15549 |  | N | Motor |  |  | 60 | 5.26 | 193 | 27.6 | 3 | 30 | 500 |
| 36 | W16/02 | 16.8607 | 96.15601 |  | N | Motor |  |  | 50 | 5.43 | 192 | 27.3 | 8 | 20 | 501 |
| 37 | W16/03 | 16.86142 | 96.15691 |  | N | Motor |  |  | 60 | 6.46 | 152 | 27 |  | 30 |  |
| 38 | W16/04 | 16.86261 | 96.15759 |  | N | Motor |  |  | 60 | 6.28 | 173 | 27 | 8 | 30 | 500 |
| 39 | W2/01 | 16.84363 | 94.82573 |  | N | Motor | no colour | Black | 130 | 6.57 | 140 | 28 | 8 | 60 |  |
| 40 | W2/02 | 16.84315 | 94.82561 |  | N | Motor |  |  | 290 | 6.91 | 138 | 28.2 | 8 | 140 |  |
| 41 | W2/03 | 16.83949 | 94.82145 |  | N | Motor | Red | black | 50 | 5.05 | 220 | 28.1 |  | 20 |  |
| 42 | W2/04 | 16.83947 | 94.8196 |  | N | Motor |  |  | 370 | 6.26 | 137 | 27.9 | 3 | 180 | 200 |
| 43 | W2/05 | 16.84001 | 94.82078 |  | N | Motor |  | black | 330 | 6.38 | 124 | 28.2 |  | 160 |  |
| 44 | W2/06 | 16.84027 | 94.81975 |  | N | Motor |  |  | 380 | 6.5 | 136 | 27.5 |  | 190 |  |
| 45 | W3/01 | 16.91693 | 94.90901 |  | N |  | Red | Red | 170 | 6.52 | 5 | 27.8 | 3 | 80 | 500 |
| 46 | W3/02 | 16.91652 | 94.91032 |  | N |  | no colour | no colour | 150 | 6.08 | 90 | 27.6 | 3 | 70 | 500 |
| 47 | W3/03 | 16.92632 | 94.89733 |  | N |  | Red | Red | 940 | 6.6 | -9 | 28.5 | 4 | 470 | 65 |
| 48 | W3/04 | 16.92187 | 94.90225 |  | N |  | Red | Red | 180 | 6.37 | -23 | 27.6 | 3.5 | 90 | 500 |
| 49 | W3/05 | 16.9344 | 94.89251 |  | N |  | Red | Black and Red | 220 | 7.05 | 46 | 26.9 | 4 | 100 | 125 |
| 50 | W3/06 | 16.93381 | 94.8933 |  | N |  | Red | Black and Red | 500 | 6.57 | -79 | 26.7 | 4 | 250 | 85 |
| 51 | W4/01 | 16.98557 | 94.99617 | Y |  | Hand Pump | |  | 1330 | 7.14 | 83 | 29 | 4 | 660 | 70 |
| 52 | W4/02 | 16.98618 | 94.99454 | Y |  | Hand Pump | Black |  | 1450 | 7.06 | 66 | 29.4 |  | 720 |  |
| 53 | W4/03 | 16.9872 | 94.9946 | Y |  | Hand Pump | |  | 1800 | 7.04 | -20 | 29.2 | 3 | 890 | 60 |
| 54 | W4/04 | 16.98601 | 94.94419 | Y |  | Motor |  |  | 240 | 7.27 | -49 | 28.9 | 8 | 110 | 120 |
| 55 | W4/05 | 16.98473 | 94.99883 |  |  | Motor |  |  | 1190 | 7.68 | -2 | 28.8 |  |  |  |
| 56 | W5/01 | 17.0322 | 95.06715 | y |  | Hand Pump | no colour | no colour | 920 | 7.1 | -79 | 27.3 | 5 | 460 | 75 |
| 57 | W5/02 | 17.07335 | 95.06701 | y |  | Hand Pump | Red | Red | 980 | 7.2 | -109 | 27.6 | 4 | 480 | 70 |
| 58 | W5/03 | 17.0741 | 95.06819 |  | N | Motor | no colour | no colour | 2420 | 7.2 | 48 | 28 | 6 | 1210 | 90 |
| 59 | W5/04 | 17.07453 | 95.06945 |  | N | Hand Pump | Red | Red | 920 | 7.02 | -102 | 28 |  | 450 |  |
| 60 | W5/05 | 17.03735 | 95.06835 |  | N | Motor |  |  | 1050 | 7.21 | 14 | 28.1 | 3 | 520 | 65 |
| 61 | W6/01 | 17.08784 | 95.17644 |  | N | Hand Pump | Red | Red | 190 | 6.84 | 140 | 27.4 | 6 | 90 | 125 |
| 62 | W6/02 | 17.09122 | 95.17912 |  | N | Hand Pump | Red | Red | 260 | 6.93 | -47 | 27.4 | 4 | 130 | 100 |
| 63 | W6/03 | 17.09087 | 95.179 |  | N | Motor | no colour | no colour | 480 | 7.19 | 117 | 27.4 | 10 | 210 | 75 |
| 64 | W6/04 | 17.09253 | 95.1903 |  | N | Motor | no colour | no colour | 360 | 7.4 | 100 | 27.4 | 8 | 180 | 80 |
| 65 | W6/05 | 17.09222 | 95.19099 |  | N | Motor | no colour | no colour | 300 | 7.35 | 141 | 28 | 8 | 140 | 80 |
| 66 | W6/06 | 17.10172 | 95.18438 | Y |  | Motor | red | black and Red | 660 | 6.85 | 60 | 28.2 |  | 3330 |  |
| 67 | W7/01 | 17.06691 | 95.28181 |  | N | Hand Pump | Red | Red | 200 | 6.69 | -88 | 28.5 | 4 | 100 | 140 |
| 68 | W7/02 | 17.06338 | 95.28181 |  | N | Hand Pump | Red | Red | 260 | 6.8 | -96 | 27.8 |  | 130 |  |
| 69 | W7/03 | 17.06084 | 95.27763 |  | N | Hand Pump | Red | Red | 280 | 6.99 | -111 | 27.4 | 2 | 130 | 90 |
| 70 | W7/04 | 17.0608 | 95.27766 |  | N | Motor | on colour | no colour | 290 | 7.63 | -10 | 27.2 | 6 | 140 | 90 |
| 71 | W7/05 | 17.06924 | 95.28281 |  | N | Hand Pump | red |  | 110 | 6.24 | 95 | 28 | 4 | 50 | 500 |
| 72 | W8/1 | 17.02781 | 95.38471 |  | N | Hand Pump | Black stained | no colour | 330 | 7.26 | 55 | 29.4 | 4 | 160 | 100 |
| 73 | W8/2 | 17.02817 | 95.38449 |  | N | Motor |  | black and Red | 320 | 7.25 | 113 | 28.6 | 5 | 150 | 90 |
| 74 | W8/3 | 17.02774 | 95.38474 |  | N | Motor | Black stained | Black stained | 340 | 7.32 | 37 | 28.5 |  | 160 |  |
| 75 | W8/4 | 17.02435 | 95.38557 |  | N | Motor |  |  | 410 | 7.18 | 109 | 28.3 |  | 200 |  |
| 76 | W8/5 | 17.02386 | 95.38426 |  | N | Motor |  |  | 380 | 7.08 | 74 | 28.2 | 3 | 180 | 125 |
| 77 | W9/01 | 16.98538 | 95.47202 |  | N | Hand Pump | No colour | no colour | 1140 | 7.03 | 71 | 28.7 | 5 | 560 | 110 |
| 78 | W9/02 | 16.98571 | 95.47219 |  | N | Hand Pump | No colour | Blackstained | 1100 | 7.04 | 27 | 28.9 |  | 550 |  |
| 79 | W9/03 | 16.98634 | 95.47279 |  | N | Motor |  |  | 1100 | 7.01 | 21 | 27.8 | 3 | 550 | 100 |
| 80 | W9/04 | 16.98633 | 95.4731 |  | N | Motor |  |  | 1100 | 7.14 | 37 | 27.9 | 2 | 550 | 100 |
| 81 | W9/05 | 16.98551 | 95.47237 |  | N | Motor |  |  | 1110 | 7.02 | -42 | 27.4 | 1 | 550 | 110 |
